# Supplementary material for: Identification of Chlamydia pneumoniae and NLRP3 inflammasome activation in Alzheimer’s disease retina
Source: Nat Commun. 2026 Jan 22;17:771. doi: 10.1038/s41467-026-68580-4 (PMC12827417; doi:10.1038/s41467-026-68580-4)
Supplement: Supplementary file 1 — Supplementary Information [file 41467_2026_68580_MOESM1_ESM.pdf]

Supplementary Information

Identification of *Chlamydia pneumoniae* and NLRP3 inflammasome activation in Alzheimer's disease retina

Supplementary Table 1. Detailed neuropathological reports of human retinal and brain donors for histological studies.
Supplementary Table 2. List of human donors whose postmortem retinas were used for various biochemical analyses.
Supplementary Table 3. List of human donors whose postmortem brains were used for mass spectrometry.
Supplementary Table 4. Demographic data on human retinal donors for protein analyses.
Supplementary Table 5. Demographic data on human brain donors for mass spectrometry analysis**.**
Supplementary Table 6. List of antibodies for immunohistochemical and biochemical analyses.
Supplementary Table 7. Mouse primer set used for RT-PCR analyses.
Supplementary Table 8. Correlation of retinal Cpn burden versus retinal and brain AD pathologies and cognition.
Supplementary Table 9. Correlation of brain Cpn burden versus brain AD pathologies and cognition.
Supplementary Table 10. Upregulated and downregulated DEPs in AD versus NC brains from *Chlamydia* interactome.
Supplementary Table 11. Upregulated and downregulated DEPs in AD versus NC retinas from *Chlamydia* interactome.
Supplementary Table 12. Pearson's correlation analyses of markers for retinal inflammasome, cell degeneration, and gliosis with retinal AD pathology markers.
Supplementary Table 13. Spearman's correlation analyses of markers for retinal inflammasome, cell degeneration, and gliosis with brain AD pathology and cognition.
Supplementary Figure 1. Extended data of Cpn inclusions in AD retina and paired-brain tissues.
Supplementary Figure 2. Distribution of retinal Cpn across subregions and correlations with brain Cpn load.
Supplementary Figure 3. Giemsa staining of mouse lung and human retinal tissues.
Supplementary Figure 4. Extended data on fluorescence in situ hybridization (FISH) in human retina.
Supplementary Figure 5. Correlations of retinal Cpn burden with retinal p-tau isoforms and brain parameters.
Supplementary Figure 6. Cell death and immune response pathways, and association with *Chlamydia* infection in the human retina and cerebral cortex.
Supplementary Figure 7. Cell death and immune response pathways, AD neuropathology, and association with *Chlamydia* infection in the cerebral cortex.
Supplementary Figure 8. Correlation of *Chlamydia* inclusion interactors with amyloid plaque and neurofibrillary tangle burden in the AD retina.
Supplementary Figure 9. Effects of Cpn infection in human neuroblastoma (SH-SY5Y) cells and AD transgenic (AD^+) mice.
Supplementary Figure 10. Extended images of retinal NLRP3 inflammasome components, early apoptosis, and cellular pyroptosis markers.
Supplementary Figure 11. Extended data on retinal gliosis and Cpn.
Supplementary Figure 12. Cpn-associated microglia in retinas from AD, MCI, and NC individuals.
Supplementary Figure 13. Prediction of brain AD pathologies by retinal *Chlamydia pneumoniae*, NLRP3, cleaved caspase-3, and $A\beta_{42}$.
Supplementary Figure 14. The AUC box plots for retinal biomarkers across all diagnostic groups.
Supplementary Figure 15. The AUC box plots for retinal biomarkers across each diagnostic groups and prediction of disease severity.

References.

Supplementary Table 1. Detailed neuropathological reports of human retinal and brain donors for histological studies.

Donor	Age at Death	Thal (A)	Braak (B)	CERAD (C)	CAA Score	Braak Stage	CDR Score	MMSE Score	APOE status	MOCA
NC1*	93	3	2	3	0	3.5	1	27	e3/e2	n.a.
NC2* [F,G]	85	2	1	2	0	1.5	0	30	e3/e3	n.a.
NC3* [F]	81	3	1	2	0	1.5	0	23	e3/e4	23
NC4* [F,G]	99	1	2	1	0	3	0	29	e3/e3	n.a.
NC5*	95	1	1	1	0	1	0	30	e3/e3	27
NC6	76	2	0	2	0	0	0	29	e3/e3	27
NC7	69	0	0	1	0	0	1	28	n.a.	n.a.
NC8	95	3	3	2	1	5	0	30	e3/e3	n.a.
NC9	95	1	0	0	0.5	1	0	30	n.a.	n.a.
NC10	98	n.a.	n.a.	n.a.	n.a.	n.a.	n.a.	n.a.	n.a.	n.a.
NC11	95	n.a.	n.a.	n.a.	n.a.	n.a.	n.a.	n.a.	n.a.	n.a.
NC12	91	n.a.	n.a.	n.a.	n.a.	n.a.	n.a.	n.a.	n.a.	n.a.
NC13	77	n.a.	n.a.	n.a.	n.a.	n.a.	n.a.	30	n.a.	n.a.
NC14	58	n.a.	n.a.	n.a.	n.a.	n.a.	n.a.	30	n.a.	n.a.
NC15	84	n.a.	n.a.	n.a.	n.a.	n.a.	n.a.	30	n.a.	n.a.
NC16	70	n.a.	n.a.	n.a.	n.a.	n.a.	n.a.	30	n.a.	n.a.
NC17	88	n.a.	n.a.	n.a.	n.a.	n.a.	n.a.	n.a.	n.a.	n.a.
NC18	74	n.a.	n.a.	n.a.	n.a.	n.a.	n.a.	n.a.	n.a.	n.a.
NC19	87	n.a.	n.a.	n.a.	n.a.	n.a.	n.a.	30	n.a.	n.a.
NC20	80	n.a.	n.a.	n.a.	n.a.	n.a.	n.a.	n.a.	n.a.	n.a.
NC21	86	n.a.	n.a.	n.a.	n.a.	n.a.	n.a.	n.a.	n.a.	n.a.
MCI1 [F,G]	86	3	1	3	0	1.5	2	15	e3/e4	26
MCI2	93	3	2	2	2	4	3	11	e3/e3	n.a.
MCI3	93	2	0	2	0	0	3	19	e3/e2	n.a.
MCI4	97	2	3	3	1	5	1	28	e3/e3	n.a.
MCI5* [F]	94	2	1	2	0	2	0.5	29	e3/e3	23
MCI6*	89	1	2	2	1	4	0.5	24	e3/e3	21
MCI7	83	2	3	2	1.5	3.5	0	26	n.a.	n.a.
MCI8	91	2	2	2	0	3	3	29	n.a.	n.a.
MCI9	98	3	3	2	2	5	2	15	n.a.	n.a.
MCI10* [F,G]	87	3	3	3	1.5	5.5	3	13	e3/e3	n.a.
MCI1*1	88	1	2	2	0	3	3	n.a.	n.a.	24
MCI12	80	3	3	2	n.a.	5	3	29	e3/e3	n.a.
MCI13	75	n.a.	n.a.	n.a.	n.a.	n.a.	n.a.	n.a.	n.a.	n.a.
MCI14	90	n.a.	n.a.	n.a.	n.a.	n.a.	n.a.	n.a.	n.a.	n.a.

Donor	Age at Death	Thal (A)	Braak (B)	CERAD (C)	CAA Score	Braak Stage	CDR Score	MMSE Score	APOE status	MOCA
MCI15*	85	0	1	3	1	1.5	0.5	n.a.	n.a.	n.a.
AD1	90	2	3	3	1	5	2	9	n.a.	n.a.
AD2	100	2	3	3	1	5.5	2	16	n.a.	n.a.
AD3	90	3	3	3	2	6	3	n.a.	n.a.	n.a.
AD4*	88	2	3	2	1	5.5	1	4	e3/e4	18
AD5* F,G	87	2	3	3	1.5	5	3	16	e3/e4	n.a.
AD6* F	70	3	3	3	1.5	5	0.5	24	n.a.	n.a.
AD7* F	77	3	3	3	1	6	2	18	e3/e4	n.a.
AD8* G	66	3	3	3	0	6	3	2	e3/e3	n.a.
AD9* F,G	81	3	3	3	1	5	3	12	e4/e4	n.a.
AD10	99	3	3	3	1.5	4	3	23	e3/e3	n.a.
AD11	85	3	3	3	1.5	5.5	3	n.a.	e3/e3	n.a.
AD12	81	3	3	3	2	6	n.a.	4	n.a.	n.a.
AD13	83	3	2	3	2	4	1	18	n.a.	n.a.
AD14	65	3	3	3	1.5	5	3	n.a.	e4/e4	n.a.
AD15	92	2	3	2	2	5	3	9	n.a.	n.a.
AD16	88	2	3	3	1.5	5	3	4	n.a.	n.a.
AD17	66	3	3	3	1.5	5	3	19	n.a.	n.a.
AD18	86	3	3	2	3	5.5	3	18	e3/e4	n.a.
AD19	88	3	3	3	1.5	5.5	1	16	e2/e3	n.a.
AD20	93	2	2	2	1.5	3.5	3	17	n.a.	n.a.
AD21	94	3	3	3	0	5.5	3	n.a.	e3/e3	n.a.
AD22	81	3	3	3	1.5	5.5	3	12	e3/e3	n.a.
AD23	90	3	3	3	1	5.5	3	n.a.	e3/e4	n.a.
AD24	90	3	2	3	1	4	3	n.a.	e3/e4	1
AD25	93	2	3	3	1	5	3	n.a.	n.a.	n.a.
AD26	79	3	3	3	1.5	5	n.a.	n.a.	n.a.	n.a.
AD27	87	3	3	3	2	5.5	3	n.a.	e3/e4	9
AD28	88	2	2	2	0	3.5	n.a.	4	n.a.	n.a.
AD29 F,G	97	3	2	3	1	3	1	26	e3/e3	n.a.
AD30	90	3	3	2	2	5	2	18	n.a.	n.a.
AD31	85	3	2	3	1	4	n.a.	n.a.	n.a.	n.a.
AD32	99	n.a.	n.a.	n.a.	n.a.	n.a.	n.a.	n.a.	n.a.	n.a.
AD33	90	n.a.	n.a.	n.a.	n.a.	n.a.	n.a.	n.a.	n.a.	n.a.
AD34	97	3	2	3	1	3	1	26	e3/e3	n.a.

AD, Alzheimer's disease dementia; APOE, apolipoprotein alleles; A (Thal), Aβ plaque score modified from Thal; B (Braak), NFT stage modified from Braak; C (CERAD), neuritic plaque score modified from CERAD; CAA, cerebral amyloid angiopathy; CDR, clinical dementia rating; FISH, fluorescence in situ hybridization; MCI, mild cognitive impairment; MMSE, mini-mental state examination; MOCA: Montreal cognitive assessment; n.a., not available; NC, normal cognition; * indicates brain tissue used for histology; retinal tissue was not available for MCI15. *F* FISH; *G* Giemsa.

Supplementary Table 2. List of human donors whose postmortem retinas were used for various biochemical analyses.

Donor	Age at Death	Thal (A)	Braak (B)	CERAD (C)	CAA Score	Braak Stage	CDR Score	MMSE Score	APOE Status	Analysis
AD1	93	2	3	3	1	5	3	n.a.	n.a.	MS/E
AD2	88	2	3	2	1	5.5	1	4	e3/e4	MS/E/WB
AD3	94	3	3	3	0	5.5	3	n.a.	e3/e3	MS/E/WB
AD4	48	3	3	3	2	5.5	n.a.	n.a.	n.a.	MS/E/WB
AD5	72	n.a.	n.a.	n.a.	n.a.	n.a.	n.a.	n.a.	n.a.	WB
AD6	100	2	3	3	0.5	5.5	2	16	n.a.	MS/E
AD7	88	1	2	2	0	3	3	n.a.	n.a.	MS/E
AD8	81	3	3	3	0	5.5	n.a.	12	n.a.	E
AD9	74	n.a.	n.a.	n.a.	n.a.	n.a.	n.a.	n.a.	n.a.	WB
AD10	95	3	3	3	2	6	n.a.	6	e3/e3	qPCR
AD11	88	n.a.	n.a.	n.a.	n.a.	n.a.	n.a.	n.a.	n.a.	qPCR
MCI1	85	0	1	3	1	1.5	0.5	n.a.	n.a.	WB
MCI2	102	1	2	1	0	3	n.a.	21	e3/e3	qPCR
NC1	81	3	1	2	0.25	1.5	0	23	e3/e4	MS/E
NC2	75	n.a.	n.a.	n.a.	n.a.	n.a.	n.a.	n.a.	n.a.	MS/E/WB
NC3	72	n.a.	n.a.	n.a.	n.a.	n.a.	n.a.	n.a.	n.a.	MS/E/WB
NC4	69	n.a.	n.a.	n.a.	n.a.	n.a.	n.a.	n.a.	n.a.	MS/E/WB
NC5	79	n.a.	n.a.	n.a.	n.a.	n.a.	n.a.	n.a.	n.a.	MS/E/WB
NC6	85	0	1	3	1	1.5	0.5	n.a.	n.a.	MS/E
NC7	76	n.a.	n.a.	n.a.	n.a.	n.a.	n.a.	n.a.	n.a.	E
NC8	90	2	1	2	2	2	n.a.	29	e3/e3	qPCR
NC9	55	n.a	n.a	n.a	n.a	n.a	n.a	n.a	n.a	qPCR

Postmortem retinas were used for mass spectrometry, ELISA, Western blot, and qPCR analyses.

Abbreviations: AD, Alzheimer's disease; APOE, apolipoprotein, A (Thal), Aβ plaque score modified from Thal; B (Braak), NFT stage modified from Braak; C (CERAD), neuritic plaque score modified from CERAD; CAA, cerebral amyloid angiopathy; CDR, clinical dementia rating; CERAD, consortium to establish a registry for Alzheimer's disease; E, ELISA, enzyme-linked immunosorbent assay; MCI, mild cognitive impairment; MMSE, mini-mental state examination; MS, mass spectrometry; n.a., not available; NC, normal cognition; qPCR, quantitative real-time PCR; WB, Western blot. Human retinal donors for MS and ELISA analyses were included in our previous publication by Koronyo *et al., Acta Neuropathologica* 2023[1].

Supplementary Table 3. List of human donors whose postmortem brains were used for mass spectrometry.

Donor	Age at Death	Plaque Stage	Braak Stage	Neuritic Plaque	MMSE Score	APOE Status
AD1	86	C	6	4	19	3/4
AD2	89	C	6	4	15	3/4
AD3	95	C	6	3	n.a.	2/3
AD4	93	C	6	4	13	3/3
AD5	88	C	5	4	10	3/3
AD6	92	C	5	4	12	3/3
AD7	86	C	5	4	n.a.	3/3
AD8	98	C	6	4	14	3/3
AD9	91	C	6	4	17	3/3
AD10	82	C	6	4	17	3/3
NC1	87	0	2	1	30	2/3
NC2	91	0	4	1	29	2/3
NC3	95	0	2	1	n.a.	3/3
NC4	90	B	3	3	18	3/3
NC5	96	0	2	1	29	2/3
NC6	94	0	1	1	27	3/3
NC7	86	0	2	2	27	3/3
NC8	91	A	2	2	29	3/3

Abbreviations: AD, Alzheimer's disease; APOE, apolipoprotein; MMSE, Mini-Mental State Examination; n.a., not available; NC, normal cognition; Plaque Stage: 0, none; A, phase I-II; B, phase III; C, phase IV-V; Neuritic Plaque scores: 4, frequent NP; 3, moderate NP; 2, sparce NP; 1, no NPs. The original human brain mass spectrometry data on this cohort were previously published in Koronyo et al., Acta Neuropathologica 2023[1].

Supplementary Table 4. Demographic data on human retinal donors for protein analyses.

**Human Donors**		**NC**	**MCI/AD**	**t**	***p***
**N= 17**		7 (4 Females, 3 Males)	10 (6 Females, 4 Males)	-	-
**Age at Death (Years)**		76.71 ± 4.44	82.30 ±14.87	0.94	0.36
**Race**		6 White, 1 Hispanic	9 White, 1 Hispanic	-	-
**Postmortem interval (Hours)**		7.48 ± 4.22	7.88 ± 4.26	0.18	0.86
**MMSE Score** (n = 4)		23.0 ± n.a.	10.70 ± 6.11	-	-
**CDR Score** (n = 8)		0.25 ± 0.35	2.08 ± 1.11	2.19	0.072
**Brain Neuropathology** Severity Score (n = 10)	Braak stage (%)	I-II (100%)	V-VI (75%)	2.76	0.025
	ABC (amyloid, Braak, CERAD)	1.67 ± 0.47	2.46 ± 0.64	1.61	0.15
	Aβ plaque	1.75 ± 0.70	2.56 ± 1.10	0.98	0.35
	NFTs	0.88 ± 0.47	2.54 ± 1.16	2.36	0.043
	NTs	0.22 ± 0.11	1.14 ± 0.75	1.67	0.13
	Atrophy	0.55 ± 0.07	1.12 ± 0.99	0.78	0.46

Protein data include mass spectrometry (MS) and ELISA re-analyses, and Western blot analysis.

Mean ABC scores were determined as follows: A, Aβ plaque score modified from Thal; B, NFT stage modified from Braak; C, neuritic plaque score modified from CERAD.

Group values are presented as mean ± standard deviation. The *t* and unadjusted *p* values were determined by two-sided unpaired Student's *t*-test.

Abbreviations: Aβ, amyloid beta; AD, Alzheimer's disease; CDR, clinical dementia rating; CERAD, consortium to establish a registry for Alzheimer's disease; MCI, mild cognitive impairment; MMSE, mini-mental state examination; NC, normal cognition; NFTs, neurofibrillary tangles; NTs, neuropil threads.

Supplementary Table 5. Demographic data on human brain donors for mass spectrometry analysis.

Human Donors		NC	AD	t	*p*
**n = 18** (Female, Male)		8 (5, 3)	10 (7, 3)	-	-
**Age at Death** [Years]		91.3 ± 3.6	90.0 ± 4.8	0.61	0.55
**Race** [No]		White (7)<br>Hispanic /Asian (1)	White (9)<br>Hispanic (1)	-	-
**MMSE Score**		27.0 ± 4.1	14.6 ± 2.9	6.74	<0.0001
**Postmortem interval** [Hours]		3.7 ± 0.8	5.3 ± 3.2	1.43	0.17
**Brain Neuropathology Severity Score** (n = 18)	**Aβ-Plaque Stage** (No; %)	None (6; 75%)<br>Stage A (1; 12.5%)<br>Stage B (1; 12.5%)	Stage C (10; 100%)	-	-
	**Braak Stage** (NO; %)	I-II (6; 75%)<br>III-IV (2; 25%)<br>V-VI (0)	V-VI (10; 100%)	-	-

Values are presented as mean ± standard deviation. The *t* and unadjusted *p* values were determined by two-sided unpaired Student's *t*-test.

Abbreviations: Aβ, Amyloid-β protein; Aβ-Plaque stages score: None, no Aβ plaque or amyloid plaque; A, mild, A1 Thal phases 1 or 2; B, moderate, A2 Thal phase 3; C, severe plaque pathology, A3 Thal phases 4 or 5; AD, Alzheimer's disease; Braak (NFT) stage scores; MMSE, mini-mental state examination; NC, normal cognition; NFT, neurofibrillary tangles.

Supplementary Table 6. List of antibodies for immunohistochemical and biochemical analyses.

Antibodies	Source species	Target species	Dilution	Application	Source	Catalog #
**Primary Antibodies**						
Cpn pAb	Rabbit	Cpn	1:500	IHC-DAB/F	MyBioSource	MBS534621
Cpn mAb	Mouse	Cpn	1:50	IHC-F	invitrogen	MA5-18183
Cpn mAb	Mouse	Cpn	1:100-200	IHC-DAB/ICC-F	invitrogen	MA5-18183`
NLRP3 mAb	Rat	Hu	1:100	IHC-F/ICC-F	R&D systems	MAB7578
NLRP3 mAb	Rabbit	Hu	1:500	WB	NovusBio	NBP2-12446
NLRP3 mAb	Rat	Hu, ms	1:1000	WB	ThermoFisher	MA5-23919
Caspase-1 mAb	Rabbit	Hu	1:150	IHC-F	R&D systems	MAB62156
ASC pAb	Rabbit	Hu, ms	1:300	IHC-F	AdipoGen	AG-25B-0006
NGSDMD mAb	Rabbit	Hu	1:500	IHC-F	Cell Signaling	36425
NGSDMD mAb	Rabbit	Hu	1:1000	WB	Cell Signaling	36425
GSDMD pAb	Rabbit	Hu, ms	1:1000	WB	MyBiosource	MBS9613058
Cleaved Caspase-3 pAb	Rabbit	Hu	1:400	IHC-F	Cell Signaling	9661
GFAP pAb	Goat	Hu, ms	1:500	IHC-F	Invitrogen	13-0300
IBA1 mAb	Rabbit	Hu, ms	1:400	IHC-F	Wako	019-19741
IBA1 mAb	Goat	Hu, ms	1:300	IHC-F	NovusBio	NB100-1028
Vimentin mAb	Rabbit	Hu	1:350	IHC-F	abcam	ab92547
NeuN mAb	Rabbit	Hu	1:500	IHC-F	abcam	ab177487
Aβ (6E10) mAb	Mouse	Hu	1:200	IHC-F	Biolegend	803001
$A\beta_{42}$ (H31L21) mAb	Rabbit	Hu, ms	1:250	ICC-F	ThermoFisher	700254
$A\beta_{42}$ (12F4) mAb	Mouse	Hu	1:1000	WB	Biolegend	805501
Aβ oligomers (scFvA13) mAb	Mouse*	Hu	1:450	IHC-F	Dr. Giovanni Meli	-
Tau Oligomers (T22) mAb	Rabbit	Hu	1:200	IHC-F	Dr. Rakez Kayed	-
$CitR_{209}$-tau mAb	Mouse	Hu	1:5000	IHC-F	Dr. Daniel Lee	-
PHF-Tau (PHF-1) mAb	Mouse	Hu	1:200	IHC-F	Dr. Peter Davies	-
Phospho-tau (Ser396) pAb	Rabbit	Hu	1:500	IHC-F	Anaspec	AS-54977
Phospho-tau (Ser202, Thr205) mAb (AT8) mAb	Mouse	Hu, ms	1:250	IHC-F	ThermoFisher	MN1020
MC-1	Mouse	Hu	1:200	IHC-F	Dr. Peter Davies	-
IL1β pAb	Rabbit	Hu	1:1000	WB	abcam	ab9722
IL1β pAb	Goat	Ms	1:100	ICC	R&D Systems	AF-401-NA
TMEM119 pAb	Rabbit	Hu, ms	1:500	IHC-F	ThermoFisher	PA5-62505
β-actin (8H10D10) mAb	Mouse	Hu, ms	1:1000	WB	Cell signaling	3700S
GAPDH (D16H11) mAb	Rabbit	Hu, ms	1:1000	WB	Cell signaling	5174
GAPDH mAb	Mouse	Hu, ms	1:1000	WB	Millipore Sigma	G8795
**Secondary Antibodies**						

Cy3 (anti-mouse, -rat, -goat, & -rabbit)	Donkey		1:200	IF	Jackson ImmunoResearch Laboratories	715-165-150, 112-165-167, 705-165-147, 711-165-152
Cy5 (anti-mouse, -rat, -goat, & -rabbit)	Donkey		1:200	IF	Jackson ImmunoResearch Laboratories	715-175-150, 712-175-153, 705-175-147, 711-175-152
Cy2 (anti-goat, & -rabbit)	Donkey		1:200	IF	Jackson ImmunoResearch Laboratories	705-225-147, 711-225-152
HRP (anti-mouse, anti-rabbit)	Goat		-	IHC/DAB	Vectastain Elite ABC Kit	PK-6102, PK-6101
Peroxidase affinipure® (anti-rat, anti-rabbit)	Goat		1:10000	WB	Jackson ImmunoResearch Laboratories	112-035-003 111-035-003
IRDye® 680RD pAb	Rabbit	Hu, ms	1:10000	WB	Licorbio	926-68071
IRDye® 800CW pAb	Rabbit	Hu, ms	1:10000	WB	Licorbio	926-32211
IRDye® 680RD pAb	Mouse	Hu, ms	1:10000	WB	Licorbio	926-68070
IRDye® 800CW pAb	Mouse	Hu, ms	1:10000	WB	Licorbio	926-32210

Abbreviations: Aβ - amyloid β-protein; ASC - apoptosis-associated speck like protein; CCasp3 - Cleaved Caspase-3; Cpn - *Chlamydia pneumoniae*; DAB - 3,3-Diaminobenzidine; GFAP - glial fibrillary acidic protein; HRP - Horseradish peroxidase; IBA1 - ionized calcium binding adaptor molecule 1; ICC - immunocytochemistry; IHC - immunohistochemistry; IHC-F - fluorescence; Hu – human; ms - mouse; mAb - monoclonal antibody; IL1β - interleukin 1 beta; NGSDMD - Gasdermin D with cleaved N-terminus; NLRP3 - nucleotide-binding oligomerization domain-like receptor containing pyrin domain 3; pAb - polyclonal antibody. PHF – paired-helical filament; scFv – single chain Fv fragment; TMEM119 - Transmembrane protein 119, WB - Western blot. *mouse recombinant antibody fragment.

Supplementary Table 7: Mouse primer set used for RT-PCR analyses.

Gene name	F/R	Sequence (5'-3')
Nlrp3	F	ATTACCCGCCCGAGAAAGG
	R	TCGCAGCAAAGATCCACACAG
Il6	F	TAGTCCTTCCTACCCCAATTTCC
	R	TTGGTCCTTAGCCACTCCTTC
Il1β	F	GCAACTGTTCCTGAACTCAACT
	R	ATCTTTTGGGGTCCGTCAACT
Gapdh	F	GTGAAGGTCGGTGTGAACG
	R	GGTCGTTGATGGCAACAATCTC

F, forward primer; R, reverse primer

Supplementary Table 8: Correlation of retinal Cpn burden versus retinal and brain AD pathologies and cognition.

**Pearson's correlation (r_p) analysis: Retinal Cpn versus retinal AD pathologies**					
$A\beta_{42}$ (12F4)	$A\beta_{40}$ (11A50-B10)	Aβ oligomers (scFvA13)	pS396-tau	PHF-tau (PHF-1)	Oligo-tau (T22)
0.63****<br>n = 39	0.65**<br>n = 21	0.18<br>n = 22	0.38*<br>n = 44	0.54**<br>n = 23	0.43**<br>n = 43
Cit-tau	AT8-tau	MC-1 tau	NLRP3	Caspase-1	ASC
0.48**<br>n = 37	0.02<br>n = 39	0.04<br>n = 43	0.70****<br>n = 27	0.87****<br>n = 27	0.60***<br>n = 27
NGSDMD	CCasp3	IBA1	GFAP	Vimentin	S100β
0.51**<br>n = 27	0.73****<br>n = 25	0.65****<br>n = 32	0.70****<br>n = 28	0.55**<br>n = 21	0.36<br>n = 6
Nissl loss	Atrophy				
-0.43*<br>n = 34	0.75****<br>n = 23				
**Spearman's correlation (r_s): Retinal Cpn versus brain AD pathologies**					
ABC	Braak Stage	Aβ plaques	NFTs	NTs	Gliosis
0.54****<br>n = 60	0.54****<br>n = 60	0.40**<br>n = 60	0.54****<br>n = 60	0.37**<br>n = 60	0.40**<br>n = 60
Atrophy	CAA				
0.48***<br>n = 60	0.35**<br>n = 60				
**Spearman's correlation (r_s): Retinal Cpn versus cognition**					
CDR	MMSE	MOCA			
0.43**<br>n = 56	-0.53****<br>n = 50	-0.56*<br>n = 15			

Pearson's or Spearman's rank correlation analyses: *r* and two-sided unadjusted *p* (asterisks) values determine the strength and statistical significance of each pairwise association between retinal Cpn burden versus retinal or brain AD pathologies and cognitive parameters. Statistics *$p < 0.05$, **$p < 0.01$, ***$p < 0.001$, ****$p < 0.0001$.

Supplementary Table 9: Correlation of brain Cpn burden versus brain AD pathologies and cognition.

**Spearman's correlation (r_s): Brain Cpn versus Brain AD pathologies**					
ABC	Braak Stage	Aβ plaques	NFTs	NTs	Gliosis
0.74**<br>n = 16	0.72**<br>n = 16	0.45<br>n = 16	0.73**<br>n = 16	0.75**<br>n = 16	0.77***<br>n = 16
Atrophy	CAA				
0.60*<br>n = 16	0.57*<br>n = 16				
**Spearman's correlation (r_s): Brain Cpn versus cognition**					
CDR	MMSE	MOCA			
0.50<br>n = 16	-0.73**<br>n = 14	-0.52<br>n = 6			

Spearman's rank correlation analyses: *r* and two-sided unadjusted *p* (asterisks) values determine the strength and statistical significance of each pairwise association between brain Cpn burden versus brain AD pathologies and cognition. Statistics *$p < 0.05$, **$p < 0.01$, ***$p < 0.001$.

Supplementary Table 10. Upregulated and downregulated DEPs in AD versus NC brains from *Chlamydia* interactome.

Accession	Symbol	Description	FC	*p*	adj. *p*	Refs
Upregulated in AD brain (32)						
Q13501	SQSTM1	Sequestosome-1	1.48	0.0001	0.0294	2,3
Q08379	GOLGA2	Golgin subfamily A member 2	1.47	0.0190	0.1605	4
O43291	SPINT2	Kunitz-type protease inhibitor 2	1.45	0.0439	0.2296	2
P08670	VIM	Vimentin	1.45	0.0013	0.0650	3
E9PND2	CSRP1	Cysteine and glycine-rich protein 1	1.37	0.0411	0.2223	4
Q8N4Q1	CHCHD4	Mitochondrial intermembrane space import and assembly protein 40	1.36	0.0462	0.2344	5
Q15796	SMAD2	Mothers against decapentaplegic homolog 2	1.36	0.0329	0.2030	4
Q9Y676	MRPS18B	28S ribosomal protein S18b, mitochondrial	1.34	0.0069	0.1104	5
O95817	BAG3	BAG family molecular chaperone regulator 3	1.34	0.0243	0.1757	3
G3V1V0	MYL6	Myosin light polypeptide 6	1.34	0.0007	0.0577	3
P06703	S100A6	Protein S100-A6	1.34	0.0167	0.1533	2
A1L188	NDUFAF8	NADH dehydrogenase [ubiquinone] 1 alpha subcomplex assembly factor 8	1.28	0.0485	0.2395	5
O75348	ATP6V1G1	V-type proton ATPase subunit G 1	1.28	0.0007	0.0577	2
Q14019	COTL1	Coactosin-like protein	1.28	0.0026	0.0819	2
P21333	FLNA	Filamin-A	1.27	0.0237	0.1753	3
Q9UBI6	GNG12	Guanine nucleotide-binding protein G(I)/G(S)/G(O) subunit gamma-12	1.27	0.0195	0.1624	2
P56385	ATP5ME	ATP synthase subunit e, mitochondrial	1.27	0.0103	0.1300	5
P04792	HSPB1	Heat shock protein beta-1	1.27	0.0082	0.1197	2,3
P04080	CSTB	Cystatin-B	1.26	0.0075	0.1151	4
P17931	LGALS3	Galectin-3	1.26	0.0027	0.0822	3
Q96HC4	PDLIM5	PDZ and LIM domain protein 5	1.25	0.0118	0.1347	4
O75369	FLNB	Filamin-B	1.25	0.0429	0.2265	4
Q6DD88	ATL3	Atlastin-3	1.24	0.0024	0.0802	2,5
J3KN67	TPM3	Tropomyosin alpha-3 chain	1.24	0.0103	0.1300	2-4
P61254	RPL26	60S ribosomal protein L26	1.22	0.0483	0.2395	4
Q15691	MAPRE1	Microtubule-associated protein RP/EB family member 1	1.22	0.0003	0.0430	5
Q12797	ASPH	Aspartyl/asparaginyl beta-hydroxylase	1.21	0.0205	0.1651	2,5
Q9BQT9	CLSTN3	Calsyntenin-3	1.21	0.0171	0.1545	5
Q9C0C2	TNKS1BP1	182 kDa tankyrase-1-binding protein	1.21	0.0054	0.1055	3
Q09666	AHNAK	Neuroblast differentiation-associated protein AHNAK	1.22	0.0342	0.2053	3,4
Q5TZA2	CROCC	Rootletin	1.22	0.0395	0.2205	2
P05556	ITGB1	Integrin beta-1	1.20	0.0065	0.1076	3
Downregulated in AD brain (52)						
Q9NZ01	TECR	Very-long-chain enoyl-CoA reductase	-2.05	0.0246	0.1759	2
Q9NQC3	RTN4	Reticulon-4	-1.83	0.0039	0.0926	2,4,5
Q9UGP8	SEC63	Translocation protein SEC63 homolog	-1.70	0.0089	0.1244	2

Q6IA86	ELP2	Elongator complex protein 2	-1.62	0.0209	0.1667	5
E9PK54	HSPA8	Heat shock cognate 71 kDa protein	-1.53	0.0411	0.2223	2
Q9BVC4	MLST8	Target of rapamycin complex subunit LST8	-1.51	0.0263	0.1803	5
O43760	SYNGR2	Synaptogyrin-2	-1.49	0.0208	0.1667	2,4
P61204	ARF3	ADP-ribosylation factor 3	-1.47	0.0128	0.1379	2
Q9P035	HACD3	Very-long-chain (3R)-3-hydroxyacyl-CoA dehydratase 3	-1.43	0.0372	0.2131	2
P61225	RAP2B	Ras-related protein Rap-2b	-1.40	0.0007	0.0577	2
Q8TCJ2	STT3B	Dolichyl-diphosphooligosaccharide--protein glycosyltransferase subunit	-1.39	0.0185	0.1593	2,5
O15258	RER1	Protein RER1	-1.37	0.0263	0.1801	2,5
O43747	AP1G1	AP-1 complex subunit gamma-1	-1.37	0.0196	0.1625	2
O95716	RAB3D	Ras-related protein Rab-3D	-1.36	0.0000	0.0270	2
P0CG08	GPR89B	Golgi pH regulator B	-1.36	0.0080	0.1194	5
K7EJH8	ACTN4	Alpha-actinin-4	-1.33	0.0062	0.1071	2,4
P63096	GNAI1	Guanine nucleotide-binding protein G(i) subunit alpha-1	-1.33	0.0034	0.0892	4
Q92905	COPS5	COP9 signalosome complex subunit 5	-1.32	0.0015	0.0689	5
O15144	ARPC2	Actin-related protein 2/3 complex subunit 2	-1.32	0.0133	0.1384	2
P07099	EPHX1	Epoxide hydrolase 1	-1.32	0.0340	0.2053	2
F2Z2X4	XPO4	Exportin-4	-1.31	0.0026	0.0819	5
Q7Z7H5	TMED4	Transmembrane emp24 domain-containing protein 4	-1.31	0.0230	0.1735	2
P15531	NME1	Nucleoside diphosphate kinase A	-1.28	0.0028	0.0825	4
Q01581	HMGCS1	Hydroxymethylglutaryl-CoA synthase, cytoplasmic	-1.28	0.0119	0.1347	5
O00410	IPO5	Importin-5	-1.28	0.0401	0.2223	5
Q93050	ATP6V0A1	V-type proton ATPase 116 kDa subunit a isoform 1	-1.27	0.0015	0.0689	2,5
Q9BXS5	AP1M1	AP-1 complex subunit mu-1	-1.27	0.0120	0.1347	4,5
Q96F07	CYFIP2	Cytoplasmic FMR1-interacting protein 2	-1.27	0.0030	0.0849	4
P42704	LRPPRC	Leucine-rich PPR motif-containing protein, mitochondrial	-1.27	0.0025	0.0816	5
P60228	EIF3E	Eukaryotic translation initiation factor 3 subunit E	-1.26	0.0438	0.2294	4
O43759	SYNGR1	Synaptogyrin-1	-1.26	0.0330	0.2033	5
F8VXU5	VPS29	Vacuolar protein sorting-associated protein 29	-1.26	0.0234	0.1749	2,4
P60981	DSTN	Destrin	-1.26	0.0406	0.2223	4
P20645	M6PR	Cation-dependent mannose-6-phosphate receptor	-1.25	0.0005	0.0508	2
E9PFW3	AP2M1	AP-2 complex subunit mu	-1.25	0.0097	0.1286	5
A0A0C4DGQ5	CAPNS1	Calpain small subunit 1	-1.25	0.0185	0.1593	2,5
Q9BTE1	DCTN5	Dynactin subunit 5	-1.25	0.0007	0.0577	5
P68104	EEF1A1	Elongation factor 1-alpha	-1.24	0.0061	0.1071	2
P61106	RAB14	Ras-related protein Rab-14	-1.24	0.0051	0.1035	2
M0QYN0	MYDGF	Myeloid-derived growth factor	-1.24	0.0056	0.1055	2
P00338	LDHA	L-lactate dehydrogenase A chain	-1.23	0.0030	0.0846	2

A0A2U3TZU2	GPI	Glucose-6-phosphate isomerase	-1.23	0.0199	0.1636	[2]
Q9NZJ7	MTCH1	Mitochondrial carrier homolog 1	-1.23	0.0097	0.1286	[5]
Q06136	KDSR	3-ketodihydrosphingosine reductase	-1.23	0.0235	0.1749	[2,5]
P20340	RAB6A	Ras-related protein Rab-6A	-1.22	0.0222	0.1717	[2]
P05388	RPLP0	60S acidic ribosomal protein P0	-1.21	0.0086	0.1228	[2]
Q5VV89	MGST3	Microsomal glutathione S-transferase 3	-1.21	0.0104	0.1304	[4,5]
O14617	AP3D1	AP-3 complex subunit delta-1	-1.21	0.0182	0.1592	[5]
P61019	RAB2A	Ras-related protein Rab-2A	-1.21	0.0142	0.1425	[2]
A0A0A0MRA8	EPB41L3	Band 4.1-like protein 3	-1.21	0.0006	0.0577	[5]
O95782	AP2A1	AP-2 complex subunit alpha-1	-1.20	0.0010	0.0620	[5]
Q08209	PPP3CA	Serine/threonine-protein phosphatase 2B catalytic subunit alpha	-1.20	0.0106	0.1304	[4,5]

FC, fold change. DEPs are defined as $|FC| \geq 1.2$ and unadjusted (*p*) < 0.05 by two-sided *t*-test; FDR-adjusted (adj. *p*) values are also shown.

Supplementary Table 11. Upregulated and downregulated DEPs in AD versus NC retinas from *Chlamydia* interactome.

Accession	Symbol	Description	FC	*p*	adj. *p*	Refs
Upregulated in AD retina (40)						
Q08554	DSC1	Desmocollin-1	1.85	0.0450	0.2499	2
P15531	NME1	Nucleoside diphosphate kinase A	1.75	0.0386	0.2360	4
P04792	HSPB1	Heat shock protein beta-1	1.70	0.0054	0.1415	2,3
Q14574	DSC3	Desmocollin-3	1.63	0.0042	0.1334	5
P67936	TPM4	Tropomyosin alpha-4 chain	1.62	0.0060	0.1415	2-4
J3KN67	TPM3	Tropomyosin alpha-3 chain	1.53	0.0174	0.1880	2-4
P61204	ARF3	ADP-ribosylation factor 3	1.52	0.0344	0.2315	2
P81605	DCD	Dermcidin	1.51	0.0276	0.2133	2
A0A0B4J2C3	TPT1	Translationally-controlled tumor protein	1.50	0.0156	0.1829	3
A0A087WYT3	PTGES3	Prostaglandin E synthase 3	1.50	0.0436	0.2468	2
P20810	CAST	Calpastatin	1.49	0.0449	0.2499	2,3
Q02413	DSG1	Desmoglein-1	1.45	0.0327	0.2272	2
H3BUF6	ATXN2L	Ataxin-2-like protein	1.39	0.0008	0.1203	3
Q01581	HMGCS1	Hydroxymethylglutaryl-CoA synthase, cytoplasmic	1.37	0.0013	0.1203	5
Q9UBE0	SAE1	SUMO-activating enzyme subunit 1	1.36	0.0261	0.2076	5
Q8TAA9	VANGL1	Vang-like protein 1	1.36	0.0230	0.1994	5
E9PAV3	NACA	Nascent polypeptide-associated complex subunit alpha, muscle-specific	1.34	0.0175	0.1882	2
O75348	ATP6V1G1	V-type proton ATPase subunit G 1	1.33	0.0451	0.2505	2
O43852	CALU	Calumenin	1.33	0.0459	0.2517	2
O60493	SNX3	Sorting nexin-3	1.32	0.0201	0.1940	4
P13797	PLS3	Plastin-3	1.32	0.0322	0.2263	4
O43399	TPD52L2	Tumor protein D54	1.32	0.0124	0.1734	2
O95817	BAG3	BAG family molecular chaperone regulator 3	1.30	0.0225	0.1977	3
P49354	FNTA	Protein farnesyltransferase/geranylgeranyltransferase type-1 subunit alpha	1.30	0.0426	0.2437	5
P48507	GCLM	Glutamate--cysteine ligase regulatory subunit	1.29	0.0106	0.1670	6
Q9NZ08	ERAP1	Endoplasmic reticulum aminopeptidase 1	1.28	0.0065	0.1447	2
A0MZ66	SHTN1	Shootin-1	1.27	0.0264	0.2084	4
P60981	DSTN	Destrin	1.27	0.0423	0.2434	4
Q32MZ4	LRRFIP1	Leucine-rich repeat flightless-interacting protein 1	1.27	0.0087	0.1594	2-5
P31946	YWHAB	14-3-3 protein beta/alpha	1.27	0.0163	0.1857	2,5
O15355	PPM1G	Protein phosphatase 1G	1.27	0.0025	0.1248	5
P54727	RAD23B	UV excision repair protein RAD23 homolog B	1.24	0.0431	0.2451	5
Q8IYD1	GSPT2	Eukaryotic peptide chain release factor GTP-binding subunit ERF3B	1.24	0.0058	0.1415	4
O75821	EIF3G	Eukaryotic translation initiation factor 3 subunit G	1.22	0.0077	0.1538	4
P98172	EFNB1	Ephrin-B1	1.21	0.0325	0.2268	5
Q13200	PSMD2	26S proteasome non-ATPase regulatory subunit 2	1.20	0.0168	0.1866	2
O14974	PPP1R12A	Protein phosphatase 1 regulatory subunit 12A	1.20	0.0013	0.1203	2
P27348	YWHAQ	14-3-3 protein theta	1.20	0.0333	0.2293	2-4

Q99614	TTC1	Tetratricopeptide repeat protein 1	1.20	0.0464	0.2529	3
Downregulated in AD retina (52)						
Q96KR6	FAM210B	Protein FAM210B, mitochondrial	-1.98	0.0101	0.1655	5
P54819	AK2	Adenylate kinase 2, mitochondrial	-1.53	0.0040	0.1310	5
Q5TZA2	CROCC	Rootletin	-1.49	0.0132	0.1744	2
Q9NQC3	RTN4	Reticulon-4	-1.48	0.0076	0.1534	2,4,5
Q8NBN7	RDH13	Retinol dehydrogenase 13	-1.46	0.0072	0.1485	5
O95159	ZFPL1	Zinc finger protein-like 1	-1.44	0.0054	0.1415	5
P61254	RPL26	60S ribosomal protein L26	-1.41	0.0324	0.2267	4
Q9Y6A9	SPCS1	Signal peptidase complex subunit 1	-1.41	0.0052	0.1403	2
Q96AG4	LRRC59	Leucine-rich repeat-containing protein 59	-1.41	0.0015	0.1203	2,3,5
O94826	TOMM70	Mitochondrial import receptor subunit TOM70	-1.40	0.0057	0.1415	5
P03928	MT-ATP8	ATP synthase protein 8	-1.38	0.0401	0.2389	2
Q9Y5A9	YTHDF2	YTH domain-containing family protein 2	-1.36	0.0140	0.1778	2
P24539	ATP5PB	ATP synthase F(0) complex subunit B1, mitochondrial	-1.35	0.0219	0.1966	5
Q8N5G0	SMIM20	Small integral membrane protein 20	-1.35	0.0099	0.1654	5
P56182	RRP1	Ribosomal RNA processing protein 1 homolog A	-1.34	0.0045	0.1349	5
O95573	ACSL3	Long-chain-fatty-acid--CoA ligase 3	-1.32	0.0092	0.1615	2
Q6ZNB6	NFXL1	NF-X1-type zinc finger protein NFXL1	-1.32	0.0080	0.1545	2
B4DR61	SEC61A1	Protein transport protein Sec61 subunit alpha isoform 1	-1.32	0.0169	0.1872	2
P35610	SOAT1	Sterol O-acyltransferase 1	-1.30	0.0051	0.1403	5
P42167	TMPO	Lamina-associated polypeptide 2, isoforms beta/gamma	-1.30	0.0139	0.1775	4
P28331	NDUFS1	NADH-ubiquinone oxidoreductase 75 kDa subunit, mitochondrial	-1.30	0.0249	0.2045	5
Q9NP73	ALG13	Putative bifunctional UDP-N-acetylglucosamine transferase and deubiquitinase ALG13	-1.30	0.0136	0.1767	5
P61026	RAB10	Ras-related protein Rab-10	-1.29	0.0048	0.1385	2
O94874	UFL1	E3 UFM1-protein ligase 1	-1.29	0.0020	0.1209	5
Q9BT22	ALG1	Chitobiosyldiphosphodolichol beta-mannosyltransferase	-1.28	0.0033	0.1287	2,5
Q8TC12	RDH11	Retinol dehydrogenase 11	-1.27	0.0182	0.1915	2
Q8TCJ2	STT3B	Dolichyl-diphosphooligosaccharide--protein glycosyltransferase subunit	-1.27	0.0100	0.1655	2,5
Q9NVH1	DNAJC11	DnaJ homolog subfamily C member 11	-1.27	0.0179	0.1898	5
H0YI09	TMT1A	Thiol Methyltransferase 1A	-1.27	0.0484	0.2563	5
Q15154	PCM1	Pericentriolar material 1 protein	-1.25	0.0143	0.1786	2
P60763	RAC3	Ras-related C3 botulinum toxin substrate 3	-1.24	0.0500	0.2602	6
P63000	RAC1	Ras-related C3 botulinum toxin substrate 1	-1.24	0.0253	0.2057	2,4,5
O94905	ERLIN2	Erlin-2	-1.24	0.0363	0.2330	2
P49755	TMED10	Transmembrane emp24 domain-containing protein 10	-1.24	0.0396	0.2380	2
O75396	SEC22B	Vesicle-trafficking protein SEC22b	-1.24	0.0062	0.1423	2
Q99567	NUP88	Nuclear pore complex protein Nup88	-1.23	0.0022	0.1211	4
P49792	RANBP2	E3 SUMO-protein ligase RanBP2	-1.23	0.0059	0.1415	4
O00592	PODXL	Podocalyxin	-1.23	0.0072	0.1485	2
Q9BWL3	C1orf43	Uncharacterized protein C1orf43	-1.23	0.0135	0.1755	5
Q9NX20	MRPL16	39S ribosomal protein L16, mitochondrial	-1.22	0.0286	0.2154	5

Q96CW1	AP2M1	AP-2 complex subunit mu	-1.22	0.0227	0.1984	5
Q96EY7	PTCD3	Pentatricopeptide repeat domain-containing protein 3, mitochondrial	-1.22	0.0193	0.1939	5
O94973	AP2A2	AP-2 complex subunit alpha-2	-1.22	0.0280	0.2139	5
O15027	SEC16A	Protein transport protein Sec16A	-1.22	0.0004	0.1203	4
Q9Y4P3	TBL2	Transducin beta-like protein 2	-1.21	0.0154	0.1829	5
Q9Y5M8	SRPRB	Signal recognition particle receptor subunit beta	-1.20	0.0163	0.1857	2
Q9NZ01	TECR	Very-long-chain enoyl-CoA reductase	-1.20	0.0093	0.1615	2
Q7Z7H5	TMED4	Transmembrane emp24 domain-containing protein 4	-1.20	0.0453	0.2508	2
Q9H0U4	RAB1B	Ras-related protein Rab-1B	-1.20	0.0476	0.2607	5
Q9H0P0	NT5C3A	Cytosolic 5'-nucleotidase 3A	-1.20	0.0127	0.1736	5
Q9Y2U8	LEMD3	Inner nuclear membrane protein Man1	-1.20	0.0015	0.1203	5
P51153	RAB13	Ras-related protein Rab-13	-1.20	0.0364	0.2334	2

FC, fold change. DEPs are defined as $|FC| \geq 1.2$ and unadjusted (*p*) < 0.05 by two-sided *t*-test; FDR-adjusted (adj. *p*) values are also shown.

Supplementary Table 12: Pearson's correlation analyses of markers for retinal inflammasome, cell degeneration, and gliosis with retinal AD pathology markers.

		**Retinal NLRP3 inflammasome**			**Retinal degeneration**		**Retinal gliosis**		
		NLRP3	ASC	Caspase-1	NGSDMD	CCasp3	IBA1	GFAP	Vimentin
**Retinal Amyloidosis**	Aβ_{42} (12F4)	0.81****<br>n = 19	0.40<br>n = 19	0.70***<br>n = 19	0.64**<br>n = 19	0.77***<br>n = 17	0.85****<br>n = 20	0.65**<br>n = 20	0.56*<br>n = 15
	Aβ oligomer (scFvA13)	0.41<br>n = 9	-0.40<br>n = 9	0.39<br>n = 9	-0.22<br>n = 8	0.32<br>n = 7	0.46<br>n = 9	-0.15<br>n = 10	0.05<br>n = 8
**Retinal Tauopathy**	PHF-tau (PHF-1)	0.30<br>n = 13	0.31<br>n = 13	0.19<br>n = 13	0.28<br>n = 13	0.30<br>n = 12	0.18<br>n = 15	0.60*<br>n = 13	0.21<br>n = 7
	pS396-tau	0.40*<br>n = 25	0.44*<br>n = 25	0.32<br>n = 25	0.38<br>n = 24	0.53**<br>n = 25	0.31<br>n = 27	0.43*<br>n = 24	0.19<br>n = 12
	Tau tangles (MC-1)	0.23<br>n = 24	-0.21<br>n = 24	-0.12<br>n = 24	-0.12<br>n = 20	0.06<br>n = 21	0.26<br>n = 26	0.14<br>n = 24	-0.06<br>n = 13
	Oligo-tau (T22)	0.70***<br>n = 24	0.44*<br>n = 24	0.60**<br>n = 24	0.77****<br>n = 23	0.80****<br>n = 23	0.69****<br>n = 27	0.60**<br>n = 24	0.60*<br>n = 14
**Retinal Gliosis**	IBA1	0.76****<br>n = 25	0.42*<br>n = 25	0.65***<br>n = 25	0.39<br>n = 24	0.69***<br>n = 23	-	0.65***<br>n = 24	0.81**<br>n = 9
	GFAP	0.91****<br>n = 23	0.71***<br>n = 23	0.77****<br>n = 23	0.68***<br>n = 26	0.85****<br>n = 21	0.65***<br>n = 24	-	0.42<br>n = 11
	Vimentin	0.84**<br>n=10	0.16<br>n = 10	0.78**<br>n = 10	0.54<br>n = 11	0.58<br>n = 8	0.81**<br>n = 9	0.42<br>n = 11	-
**Retinal NLRP3 Inflammasome**	NLRP3	-	0.58**<br>n = 27	0.83****<br>n = 27	0.74****<br>n = 23	0.80****<br>n = 23	0.77****<br>n = 25	0.91****<br>n = 23	0.84**<br>n = 10
	ASC	0.58**<br>n = 27	-	0.70****<br>n = 27	0.58**<br>n = 23	0.76****<br>n = 23	0.42*<br>n = 25	0.71***<br>n = 23	0.16<br>n = 10
	Caspase-1	0.83****<br>n = 27	0.70****<br>n = 27	-	0.57**<br>n = 23	0.80****<br>n = 23	0.65***<br>n = 25	0.77****<br>n = 23	0.78**<br>n = 10
**Retinal Degeneration**	NGSDMD	0.74****<br>n = 23	0.58**<br>n = 23	0.57**<br>n = 23	-	0.75****<br>n = 21	0.39<br>n = 24	0.68***<br>n = 26	0.54<br>n = 11
	CCasp3	0.81****<br>n = 23	0.76****<br>n = 23	0.80****<br>n = 23	0.75****<br>n = 21	-	0.69***<br>n = 23	0.85****<br>n = 21	0.58<br>n = 8
	Atrophy	0.86****<br>n = 14	0.62*<br>n = 14	0.82***<br>n=14	0.60*<br>n = 14	0.86***<br>n = 11	0.72**<br>n = 15	0.72**<br>n = 15	0.87*<br>n = 6

Pearson's correlation analyses: *r* and two-sided unadjusted *p* (asterisks) values determine the strength and statistical significance of each pairwise association between markers of retinal inflammasome, degeneration, and gliosis versus retinal AD-associated pathologies, inflammasomes, gliosis, and degeneration markers. Statistics $*p < 0.05$, $**p < 0.01$, $***p < 0.001$, $****p < 0.0001$.

Supplementary Table 13: Spearman's correlation analyses of markers for retinal inflammasome, cell degeneration, and gliosis with brain AD pathology and cognition.

	**Retinal inflammasome**			**Retinal degeneration**		**Retinal gliosis**		
	NLRP3	ASC	Caspase-1	NGSDMD	CCasp3	IBA1	GFAP	Vimentin
**Brain Aβ plaque**	0.35<br>n = 27	0.25<br>n = 27	0.34<br>n = 27	0.21<br>n = 25	0.42*<br>n = 25	0.28<br>n = 29	0.39*<br>n = 26	0.38<br>n = 18
**ABC**	0.68****<br>n = 27	0.35<br>n = 27	0.56**<br>n = 27	0.43*<br>n = 25	0.72****<br>n = 25	0.49**<br>n = 29	0.71****<br>n = 26	0.62**<br>n = 18
**NFTs**	0.66***<br>n = 27	0.53**<br>n = 27	0.65***<br>n = 27	0.49*<br>n = 25	0.78****<br>n = 25	0.56**<br>n = 29	0.59**<br>n = 26	0.43<br>n = 18
**Braak**	0.69****<br>n = 27	0.60***<br>n = 27	0.63***<br>n = 27	0.55**<br>n = 25	0.72****<br>n = 25	0.49**<br>n = 30	0.78****<br>n = 26	0.46<br>n = 18
**Brain atrophy**	0.47*<br>n = 27	0.27<br>n = 27	0.41*<br>n = 27	0.22<br>n = 25	0.45*<br>n = 25	0.36<br>n = 29	0.45*<br>n = 26	0.16<br>n = 18
**CAA**	0.55**<br>n = 27	0.31<br>n = 27	0.47*<br>n = 27	0.29<br>n = 25	0.59**<br>n = 25	0.49**<br>n = 29	0.39<br>n = 26	0.35<br>n = 17
**CDR**	0.67***<br>n = 27	0.26<br>n = 27	0.55**<br>n = 27	0.37<br>n = 25	0.62**<br>n = 25	0.66***<br>n = 28	0.51**<br>n = 26	0.68**<br>n = 16
**MMSE**	-0.66***<br>n = 25	-0.49*<br>n = 25	-0.58**<br>n = 25	-0.50*<br>n = 25	-0.69***<br>n = 22	-0.58**<br>n = 26	-0.48*<br>n = 26	-0.59**<br>n = 18
**MOCA**	-0.76<br>n = 7	-0.33<br>n = 7	-0.60<br>n = 7	-0.41<br>n = 6	-0.45<br>n = 8	-0.31<br>n = 7	-0.49<br>n = 6	-0.80<br>n = 4

Spearman's rank correlation analyses: *r* and two-sided unadjusted *p* (asterisks) values determine the strength and statistical significance of each pairwise association between markers of retinal inflammasome, degeneration, and gliosis versus brain AD-pathology markers. Statistics *$p < 0.05$, **$p < 0.01$, ***$p < 0.001$, ****$p < 0.0001$.

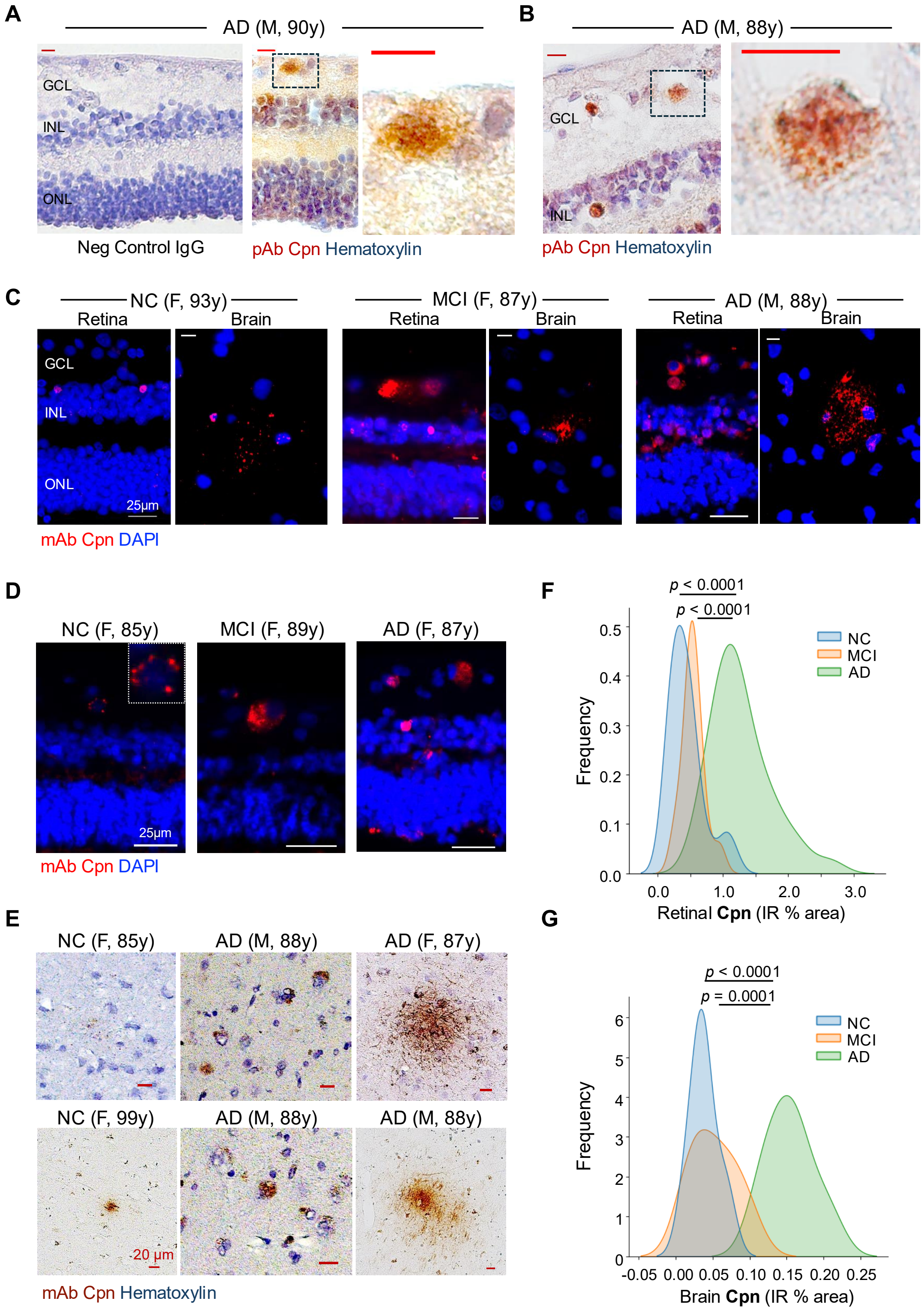
A
AD (M, 90y)
GCL
INL
ONL
Neg Control IgG
pAb Cpn Hematoxylin
B
AD (M, 88y)
GCL
INL
pAb Cpn Hematoxylin
C
NC (F, 93y)
Retina
Brain
MCI (F, 87y)
Retina
Brain
AD (M, 88y)
Retina
Brain
GCL
INL
ONL
25µm
mAb Cpn DAPI
D
NC (F, 85y)
MCI (F, 89y)
AD (F, 87y)
25µm
mAb Cpn DAPI
E
NC (F, 85y)
AD (M, 88y)
AD (F, 87y)
NC (F, 99y)
AD (M, 88y)
AD (M, 88y)
20 µm
mAb Cpn Hematoxylin
F
p < 0.0001
p < 0.0001
NC
MCI
AD
Frequency
0.0
0.1
0.2
0.3
0.4
0.5
0.0
1.0
2.0
3.0
Retinal Cpn (IR % area)
G
p < 0.0001
p = 0.0001
NC
MCI
AD
Frequency
0
1
2
3
4
5
6
-0.05
0.00
0.05
0.10
0.15
0.20
0.25
Brain Cpn (IR % area)

Supplementary Figure 1. Extended data of Cpn inclusions in AD retina and paired-brain tissues. **A**, **B** Representative peroxidase-based images (DAB brown, hematoxylin nuclei blue) of retinal cross-sections from AD patients. **A** Left image exhibit no immunoreaction when using the negative (Neg) control IgG, whereas staining with *Chlamydia pneumoniae* (Cpn, brown) polyclonal antibody (pAb) depicts the presence of Cpn inclusions in GCL and INL (middle image). Cpn inclusions in higher magnification are shown (right image). **B** AD retinal cross sections depict the presence of Cpn inclusions in the GCL and INL. Higher magnification, right image. Scale bars, 10 µm. **C** Representative fluorescence images of retinal and paired-brain (Area 9 located in the dorsolateral prefrontal cortex) cross-sections from MCI and AD patients versus NC individuals, depicting the presence of Cpn inclusions (red), with specific Cpn monoclonal antibody (mAb). DAPI (blue) stained nuclei. Scale bars, 25 µm (retina) and 10 µm (brain cross-sections). **D** Representative fluorescence images of retinal cross-sections from MCI and AD patients versus NC individuals (the same subjects which were stained using peroxidase-based (DAB) method, see Fig. 1E**)**, depicting the presence of Cpn inclusions (red), stained with specific Cpn mAb. DAPI (blue) stained nuclei. Scale bars, 25 µm. **E** Representative peroxidase-based (DAB) images of brain cross-sections from AD patients versus NC individuals showing the presence of Cpn inclusions (brown) stained with Cpn mAb and hematoxylin. Scale bars, 20 µm**. F, G** Extended data of Figure 1F: Gaussian distribution curves displaying the frequency of **(F)** retinal (n = 21 NC, 14 MCI, 34 AD) and **(G)** brain (n = 5 NC, 5 MCI, 6 AD) Cpn % IR area. M-male, F-female, and age (y) are shown. Statistics: Data from individual subjects (circles) and group means ± SEMs are shown. *p* values are by one-way ANOVA and Tukey's post hoc multiple comparison test. Ganglion cell layer (GCL); Inner nuclear layer (INL); Outer nuclear layer (ONL). Source data are provided as a Source Data file.

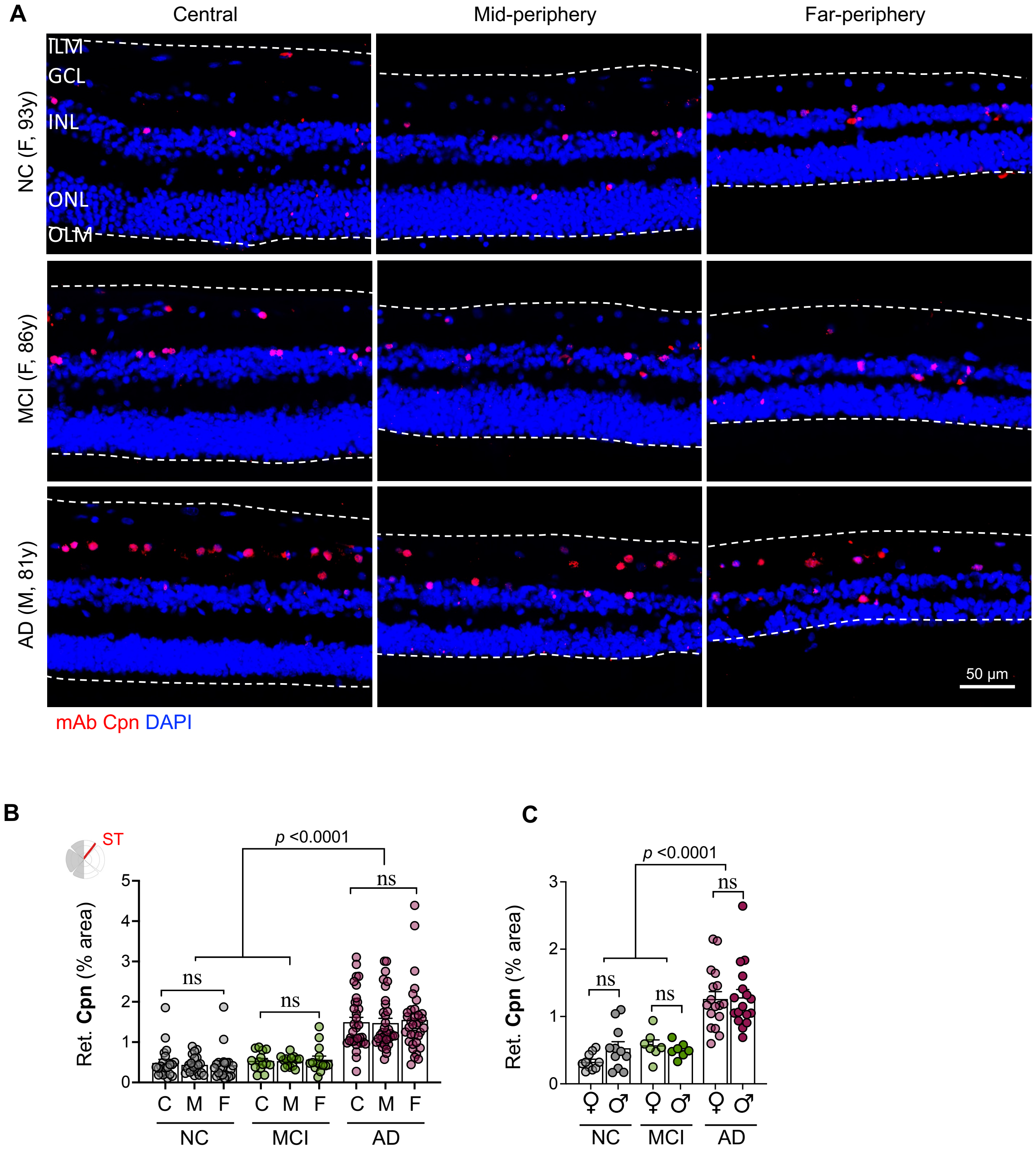

A
Central
Mid-periphery
Far-periphery
NC (F, 93y)
MCI (F, 86y)
AD (M, 81y)
ILM
GCL
INL
ONL
OLM
50 µm
mAb Cpn DAPI
B
ST
p <0.0001
ns
Ret. Cpn (% area)
C M F C M F C M F
NC
MCI
AD
C
p <0.0001
ns
Ret. Cpn (% area)
♀ ♂ ♀ ♂ ♀ ♂
NC
MCI
AD

Supplementary Figure 2. Distribution of retinal Cpn across subregions and correlations with brain Cpn load.

A Representative fluorescence images of retinal cross-sections from MCI and AD patients versus NC individuals depicting the distribution of *Chlamydia pneumoniae* (Cpn; red) inclusions across three retinal subregions (Central, Mid-periphery, and Far-periphery). DAPI (blue) stained nuclei. White dotted lines display the analyzed area, between the inner limiting membrane (ILM) and outer limiting membrane (OLM). Scale bar, 50 µm. **B, C** Scatter plots showing quantitative IHC analysis of retinal Cpn % IR area in (**B**) three retinal subregions (C, Central; M, Mid-periphery and F, Far-periphery) among NC (n=21), MCI (n=14), and AD (n=34) subjects, and (**C**) male and female subjects of the same cohort (NC=10M, 11F; MCI=7M, 7F; AD=17M, 17F). M-male, F-female, and age (y) are shown. Statistics: Data from individual subjects (circles) and group means ± SEMs are shown. ♀ = female; ♂ = Male. *p* values are by two-way ANOVA and Šídák's post hoc multiple-comparison test. ns = non-significant. Ganglion cell layer (GCL); Inner limiting membrane (ILM); Inner nuclear layer (INL); Outer limiting membrane (OLM); Outer nuclear layer (ONL). Source data are provided as a Source Data file.

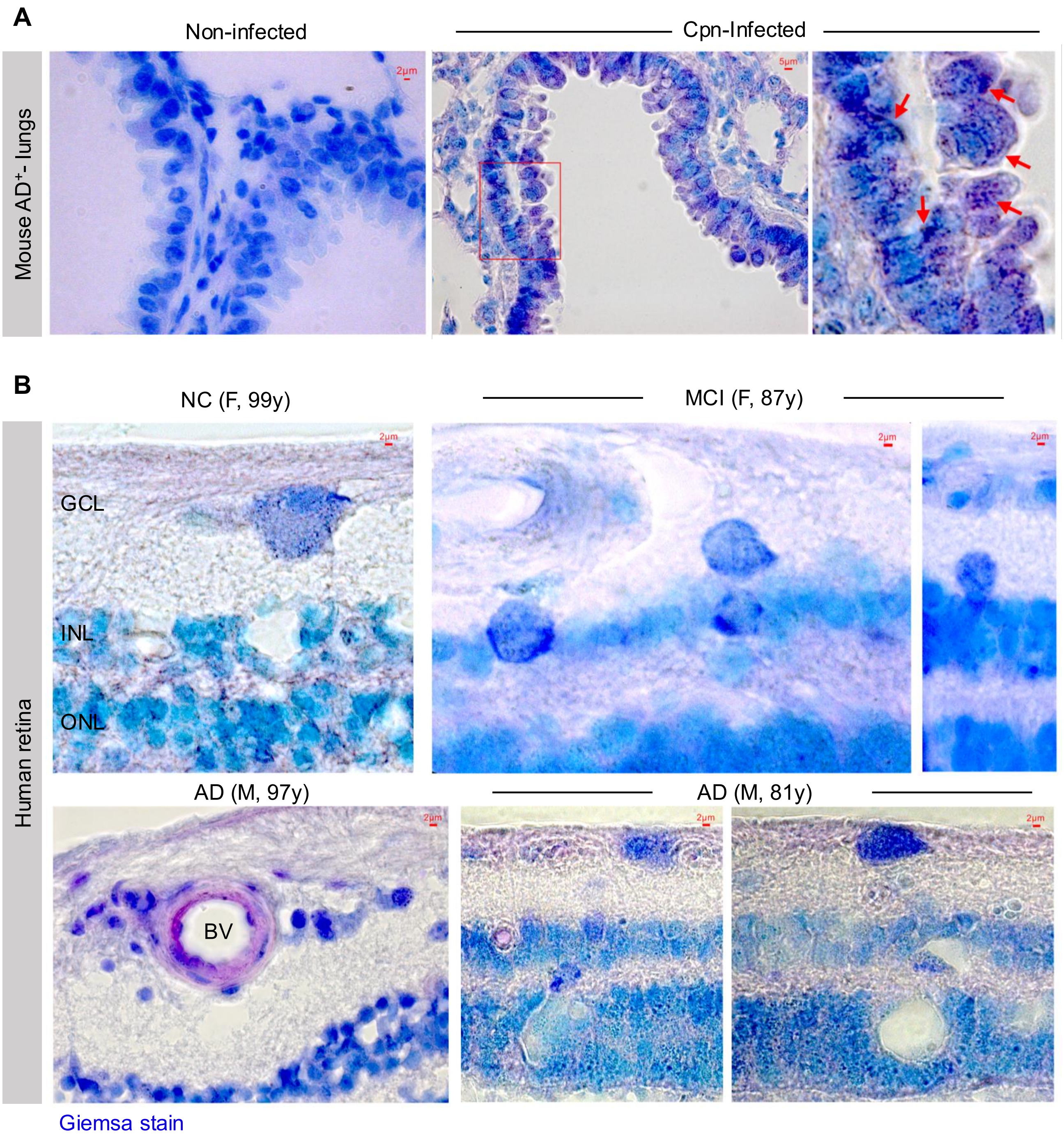

Supplementary Figure 3. Giemsa staining of mouse lung and human retinal tissues.

A Representative images of lung tissues from uninfected and infected AD-transgenic (AD^+) mice showing *Chlamydia pneumoniae* (Cpn)–like inclusion bodies (few are indicated by red arrows). **B** Extended images of Giemsa-stained human retinal cross-sections from NC, MCI, and AD subjects, demonstrating inclusion bodies morphologically comparable to those observed in infected mouse lung tissues. M-male, F-female, and age (y) are shown. Scale bars, 2-5 µm.

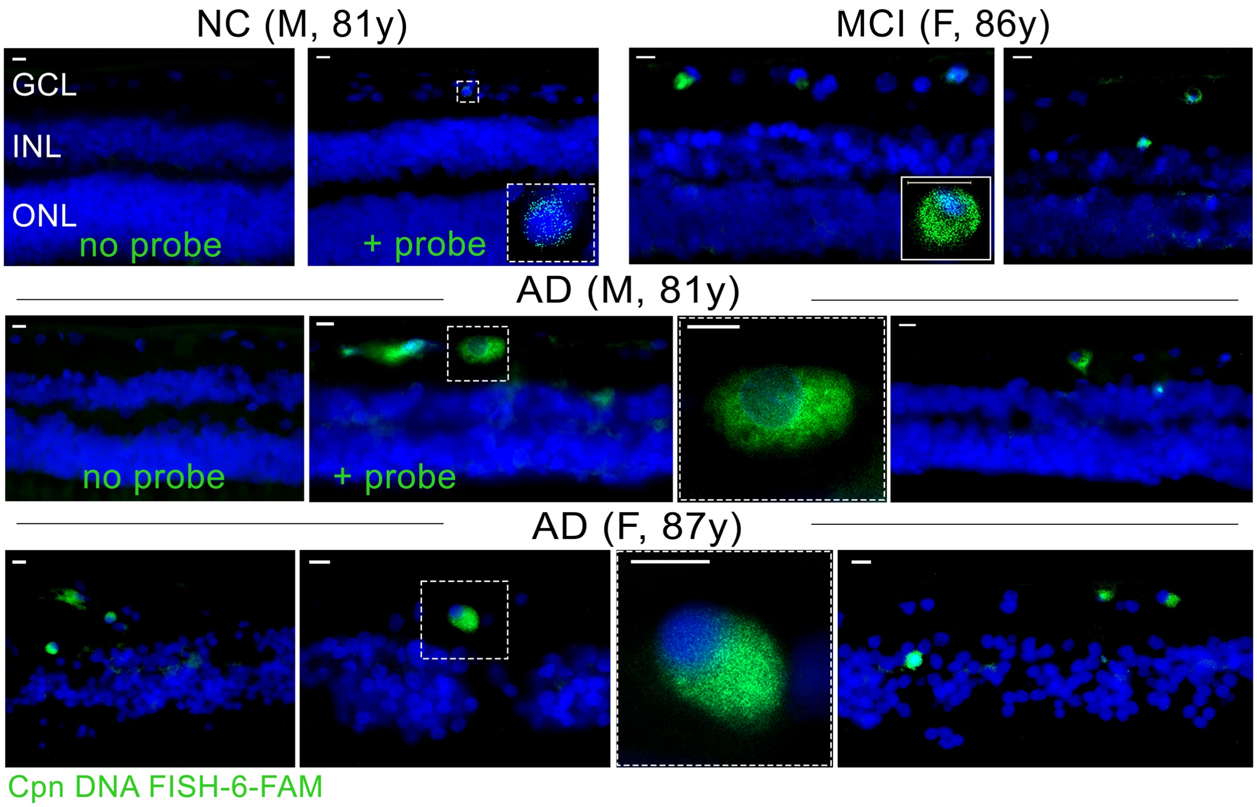

Supplementary Figure 4. Extended data on fluorescence in situ hybridization (FISH) in human retina. FISH staining was used to detect *Chlamydia pneumoniae* (Cpn) genomic DNA (green) within retinal cross-sections of MCI and AD versus NC donors, confirming bacterial localization in host cells. No probe controls were also applied on NC and AD retinas, showing no signal. M-male, F-female, and age (y) are shown. Scale bars, 10 µm.

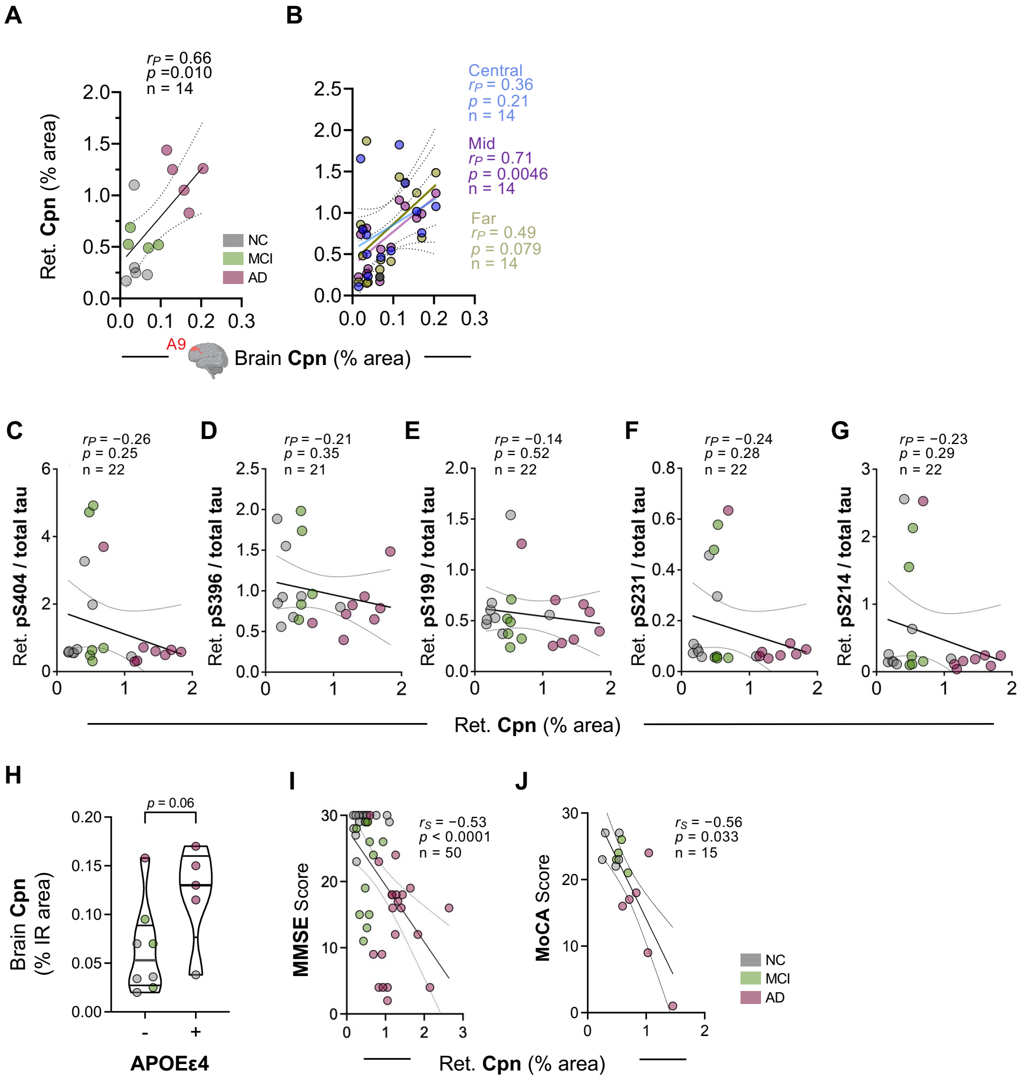

Supplementary Figure 5. Correlations of retinal Cpn burden with retinal p-tau isoforms and brain parameters.

A, B Pearson's correlation (r_p) analyses of (**A**) total retinal Cpn burden and (**B**) in C/M/F subregions with brain Cpn load (n = 14; one case who was a statistical outlier for retinal Cpn burden is excluded). **C-G** Pearson's correlation (r_p) analyses between retinal *Chlamydia pneumoniae* (Cpn) burden and the ratios of (**C**) pS404, (**D**) pS396, (**E**) pS199, (**F**) pS231, and (**G**) pS214 tau isoforms to total tau, quantified using NanoString GeoMx® Digital Spatial Profiling, as previously described[7]. **H** Brain Cpn % IR area in donors stratified based on APOEε4 genotype, in carriers (+, n = 8) vs. noncarriers (-, n = 5) subjects, as assessed by two-sided unpaired *t*-test. **I, J** Spearman's correlation (r_S) analyses of retinal Cpn burden with (**I**) MMSE and (**J**) MOCA cognitive scores. Statistics: Data from individual subjects (circles) and group means ± SEM are shown. Source data are provided as a Source Data file. Brain icone is from Biorender.com.

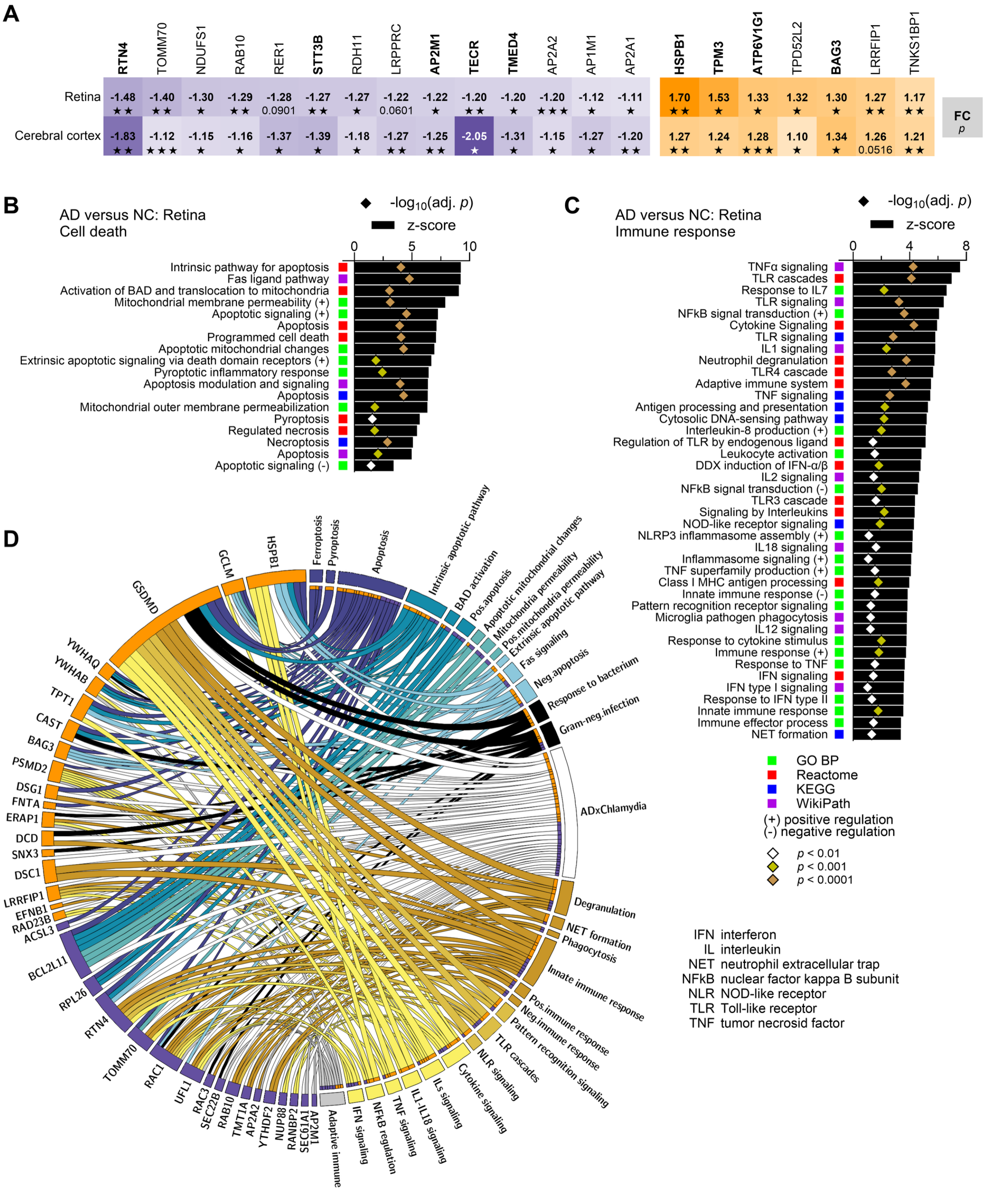
A
RTN4 TOMM70 NDUFS1 RAB10 RER1 STT3B RDH11 LRPPRC AP2M1 TECR TMED4 AP2A2 AP1M1 AP2A1 HSPB1 TPM3 ATP6V1G1 TPD52L2 BAG3 LRRFIP1 TNKS1BP1
Retina -1.48 -1.40 -1.30 -1.29 -1.28 -1.27 -1.27 -1.22 -1.22 -1.20 -1.20 -1.20 -1.12 -1.11 1.70 1.53 1.33 1.32 1.30 1.27 1.17
0.0901 0.0601
Cerebral cortex -1.83 -1.12 -1.15 -1.16 -1.37 -1.39 -1.18 -1.27 -1.25 -2.05 -1.31 -1.15 -1.27 -1.20 1.27 1.24 1.28 1.10 1.34 1.26 1.21
0.0516
FC
p
B
AD versus NC: Retina
Cell death
-log10(adj. p)
z-score
0 5 10
Intrinsic pathway for apoptosis
Fas ligand pathway
Activation of BAD and translocation to mitochondria
Mitochondrial membrane permeability (+)
Apoptotic signaling (+)
Apoptosis
Programmed cell death
Apoptotic mitochondrial changes
Extrinsic apoptotic signaling via death domain receptors (+)
Pyroptotic inflammatory response
Apoptosis modulation and signaling
Apoptosis
Mitochondrial outer membrane permeabilization
Pyroptosis
Regulated necrosis
Necroptosis
Apoptosis
Apoptotic signaling (-)
C
AD versus NC: Retina
Immune response
-log10(adj. p)
z-score
0 4 8
TNFα signaling
TLR cascades
Response to IL7
TLR signaling
NFkB signal transduction (+)
Cytokine Signaling
TLR signaling
IL1 signaling
Neutrophil degranulation
TLR4 cascade
Adaptive immune system
TNF signaling
Antigen processing and presentation
Cytosolic DNA-sensing pathway
Interleukin-8 production (+)
Regulation of TLR by endogenous ligand
Leukocyte activation
DDX induction of IFN-α/β
IL2 signaling
NFkB signal transduction (-)
TLR3 cascade
Signaling by Interleukins
NOD-like receptor signaling
NLRP3 inflammasome assembly (+)
IL18 signaling
Inflammasome signaling (+)
TNF superfamily production (+)
Class I MHC antigen processing
Innate immune response (-)
Pattern recognition receptor signaling
Microglia pathogen phagocytosis
IL12 signaling
Response to cytokine stimulus
Immune response (+)
Response to TNF
IFN signaling
IFN type I signaling
Response to IFN type II
Innate immune response
Immune effector process
NET formation
GO BP
Reactome
KEGG
WikiPath
(+) positive regulation
(-) negative regulation
p < 0.01
p < 0.001
p < 0.0001
IFN interferon
IL interleukin
NET neutrophil extracellular trap
NFkB nuclear factor kappa B subunit
NLR NOD-like receptor
TLR Toll-like receptor
TNF tumor necrosid factor
D
GSDMD GCLM HSPB1 Ferroptosis Pyroptosis Apoptosis
Intrinsic apoptotic pathway
BAD activation
Pos.apoptosis
Apoptotic mitochondrial changes
Mitochondria permeability
Pos.mitochondria permeability
Extrinsic apoptotic pathway
Fas signaling
Neg.apoptosis
Response to bacterium
Gram-neg.infection
ADxChlamydia
Degranulation
NET formation
Phagocytosis
Innate immune response
Pos.immune response
Neg.immune response
Pattern recognition signaling
TLR cascades
NLR signaling
Cytokine signaling
ILs signaling
IL1-IL18 signaling
TNF signaling
NFkB regulation
IFN signaling
Adaptive immune
AP2M1 SEC61A1 RANBP2 NUP88 YTHDF2 AP2A2 TMT1A RAB10 SEC22B RAC3 UFL1 RAC1 TOMM70 RTN4 RPL26 BCL2L11 ACSL3 RAD23B EFNB1 LRRFIP1 DSC1 SNX3 DCD ERAP1 FNTA DSG1 PSMD2 BAG3 CAST TPT1 YWHAB YWHAQ

Supplementary Figure 6. Cell death and immune response pathways, and association with *Chlamydia* infection in the human retina and cerebral cortex.

A Heatmaps of shared *Chlamydia* interactome differentially expressed proteins (DEPs) in AD retina and cerebral cortex [Fold Change (FC) and unadjusted *p*, by Pearson's correlation analysis]. The expression of 5 downregulated (RTN4, STT3B, AP2M1, TECR, TMED4) and 5 upregulated (HSPB1, TPM3, LRRFIP1 [t-test], BAG3, ATP6V1G1) proteins were within cutoffs for DEPs (|FC| > 1.2 and unadjusted $p<0.05$) in both the retina and the cortex. Additional proteins, whose expression was within cutoffs for DEPs (|FC| > 1.2 and unadjusted $p<0.05$) for either the retina or the cortex, were found. *$p < 0.05$, **$p < 0.01$, and ***$p < 0.001$, by two-sided unadjusted *t*-test. **B, C** Gene ontology (GO) analysis of DEPs related to (**B)** cell death and (**C**) immune response in human AD (n = 6) versus NC retina (n = 6). The analysis was carried out in Metascape and included the GO Biological Processes (BP), Reactome, Kyoto Encyclopedia of Genes and Genomes (KEGG) and WikiPathways databases. Bar and symbol graphs represent z-scores and Benjamini-Hochberg adjusted *p*-values from Metascape analysis, respectively. Range of *p*-values are presented as color-coded symbols. **D** Chord diagram displays the association of *Chlamydia*-interacting proteins with pathways related to cell death (blue gradient outer segments and ribbons), immune response (yellow gradient and grey outer segments and ribbons), as well as Gram (-) bacterial infection (white outer segment and ribbons). For proteins, orange and purple outer segments indicates upregulation and downregulation, respectively. Source data are provided as a Source Data file.

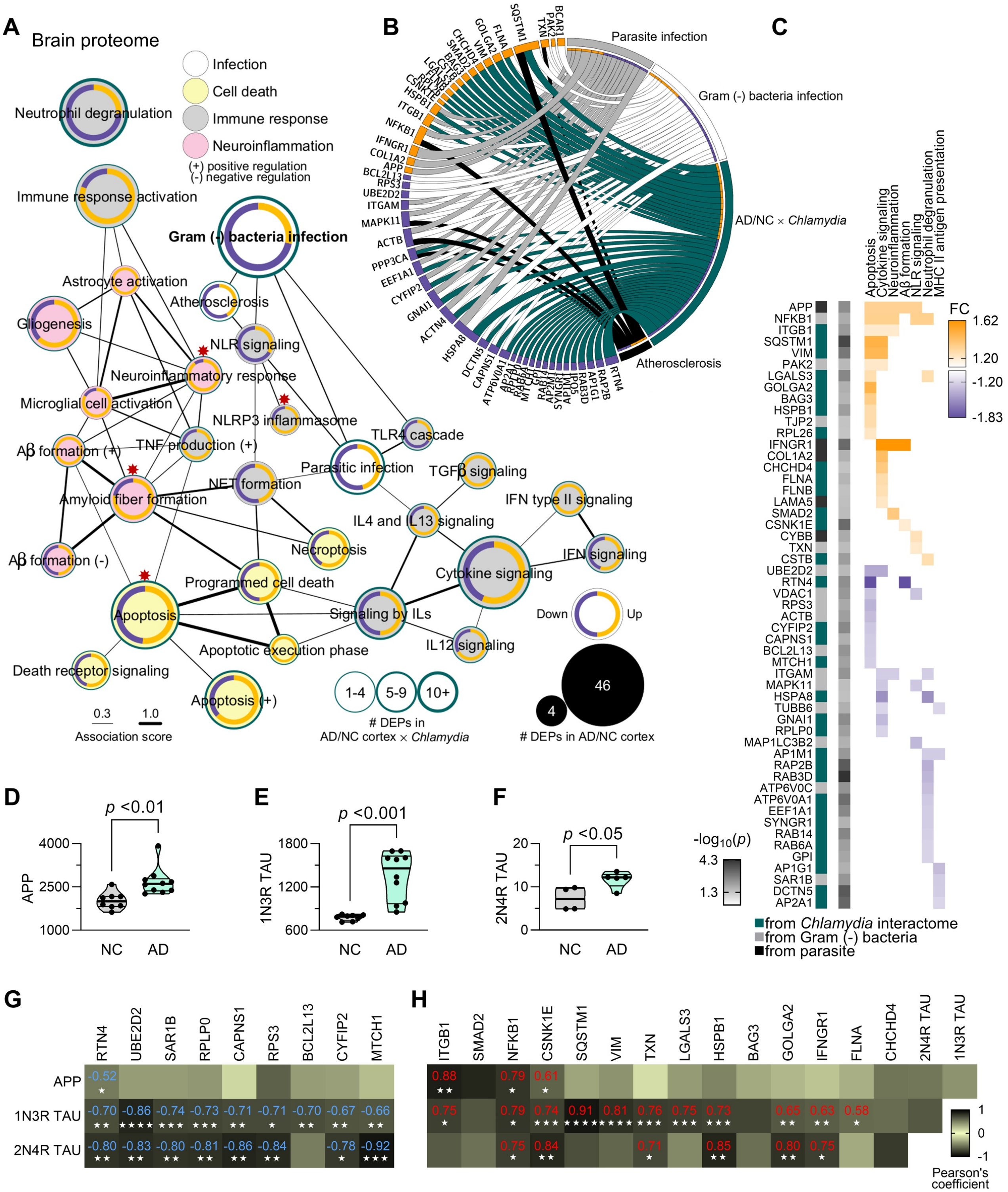
A
Brain proteome
Infection
Cell death
Immune response
Neuroinflammation
(+) positive regulation
(-) negative regulation
Neutrophil degranulation
Immune response activation
Gram (-) bacteria infection
Astrocyte activation
Atherosclerosis
Gliogenesis
NLR signaling
Neuroinflammatory response
Microglial cell activation
NLRP3 inflammasome
TLR4 cascade
Aβ formation (+)
TNF production (+)
Parasitic infection
TGFβ signaling
NET formation
Amyloid fiber formation
IFN type II signaling
IL4 and IL13 signaling
Necroptosis
IFN signaling
Aβ formation (-)
Cytokine signaling
Programmed cell death
Apoptosis
Signaling by ILs
Down
Up
Apoptotic execution phase
IL12 signaling
Death receptor signaling
Apoptosis (+)
0.3
1.0
Association score
1-4
5-9
10+
DEPs in AD/NC cortex × Chlamydia
46
4
DEPs in AD/NC cortex
B
Parasite infection
Gram (-) bacteria infection
AD/NC × Chlamydia
Atherosclerosis
C
Apoptosis
Cytokine signaling
Neuroinflammation
Aβ formation
NLR signaling
Neutrophil degranulation
MHC II antigen presentation
FC
1.62
1.20
-1.20
-1.83
APP
NFKB1
ITGB1
SQSTM1
VIM
PAK2
LGALS3
GOLGA2
BAG3
HSPB1
TJP2
RPL26
IFNGR1
COL1A2
CHCHD4
FLNA
FLNB
LAMA5
SMAD2
CSNK1E
CYBB
TXN
CSTB
UBE2D2
RTN4
VDAC1
RPS3
ACTB
CYFIP2
CAPNS1
BCL2L13
MTCH1
ITGAM
MAPK11
HSPA8
TUBB6
GNAI1
RPLP0
MAP1LC3B2
AP1M1
RAP2B
RAB3D
ATP6V0C
ATP6V0A1
EEF1A1
SYNGR1
RAB14
RAB6A
GPI
AP1G1
SAR1B
DCTN5
AP2A1
-log10(p)
4.3
1.3
from Chlamydia interactome
from Gram (-) bacteria
from parasite
D
p <0.01
APP
4000
2500
1000
NC
AD
E
p <0.001
1N3R TAU
1800
1200
600
NC
AD
F
p <0.05
2N4R TAU
20
10
0
NC
AD
G
RTN4
UBE2D2
SAR1B
RPLP0
CAPNS1
RPS3
BCL2L13
CYFIP2
MTCH1
APP
1N3R TAU
2N4R TAU
-0.52
-0.70 -0.86 -0.74 -0.73 -0.71 -0.71 -0.70 -0.67 -0.66
-0.80 -0.83 -0.80 -0.81 -0.86 -0.84 -0.78 -0.92
H
ITGB1
SMAD2
NFKB1
CSNK1E
SQSTM1
VIM
TXN
LGALS3
HSPB1
BAG3
GOLGA2
IFNGR1
FLNA
CHCHD4
2N4R TAU
1N3R TAU
0.88 0.79 0.61
0.75 0.79 0.74 0.91 0.81 0.76 0.75 0.73 0.65 0.63 0.58
0.75 0.84 0.71 0.85 0.80 0.75
1
0
-1
Pearson's coefficient

Supplementary Figure 7. Cell death and immune response pathways, AD neuropathology, and association with *Chlamydia* infection in the cerebral cortex.

A GO network (Metascape) of pathways related to infection, cell death, immune response and neuroinflammation. The size of the nodes represents the number of DEPs in AD versus NC cortex, with the inner ring showing the proportion of these DEPs that are downregulated (purple) or upregulated (orange) in AD. The green border and its thickness represent the number of DEPs that interact with *Chlamydia* inclusion in each pathway. The thickness of edges represents the shared DEPs (association score) between pathways. Red asterisks indicate pathways (NLRP3 inflammasome, apoptosis, neuroinflammatory response, and amyloid fiber formation) that were further explored and validated. **B** Chord diagram displays the association between cell death, immune response-related DEPs in AD cortex and *Chlamydia* (interactome), Gram (-) bacteria and parasitic infections, and atherosclerosis. For DEPs, orange and purple outer segments indicate upregulation and downregulation, respectively. **C** Heatmaps of DEPs [FC and $-\log_{10}(p)$] in AD cortex for selected pathways. Only proteins connected to Gram (-) bacteria and parasitic infections (Metascape analysis) and *Chlamydia* infection (*Chlamydia* interactome) are shown for each pathway. **D-F** Mass spectrometry (MS) quantitation of (**D**) Amyloid beta precursor protein (APP), (**E**) one N-terminal domain and three microtubule-binding repeat domains (1N3R) and (**F**) two N-terminal domains and four microtubule-binding repeat domains (2N4R) isoforms of TAU in cerebral cortex of human AD (n = 10) and NC retina (n = 8); Statistics: *p* values by two-sided unpaired *t*-test. **G, H** Heatmaps of (**G**) negative and (**H**) positive Pearson's correlation coefficients analysis (*r*) between *Chlamydia* interactors and APP, 1N3R TAU, and 2N4R TAU, quantified by MS. Correlation statistics: unadjusted $*p<0.05$, $**p<0.01$, $***p<0.001$, and $****p<0.0001$. Source data are provided as a Source Data file.

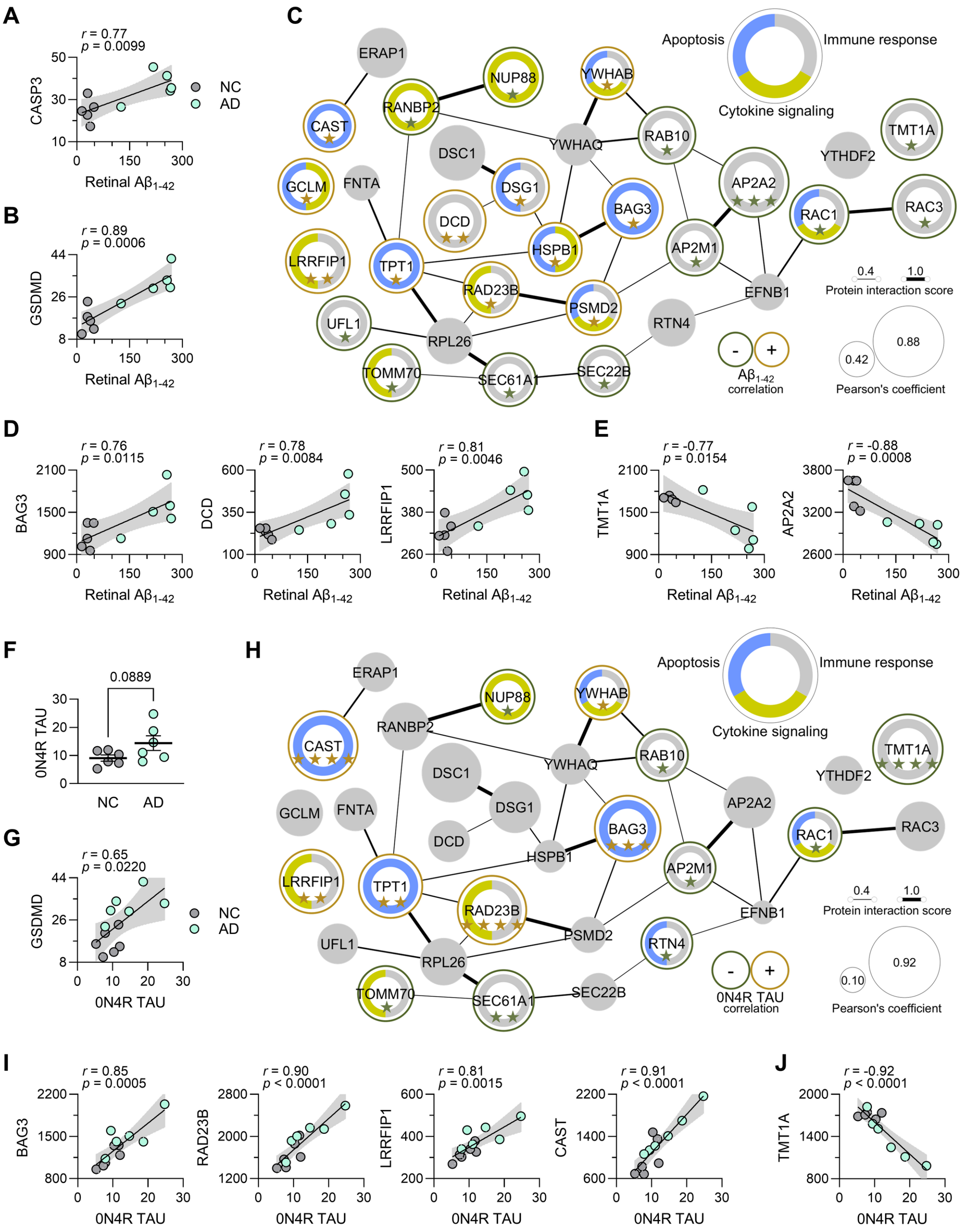
A
r = 0.77
p = 0.0099
CASP3
NC
AD
Retinal Aβ1-42
B
r = 0.89
p = 0.0006
GSDMD
Retinal Aβ1-42
C
ERAP1
NUP88
YWHAB
Apoptosis
Immune response
Cytokine signaling
RANBP2
CAST
YWHAQ
RAB10
DSC1
TMT1A
YTHDF2
GCLM
FNTA
DSG1
AP2A2
BAG3
DCD
RAC1
RAC3
HSPB1
AP2M1
LRRFIP1
TPT1
RAD23B
EFNB1
PSMD2
RTN4
UFL1
RPL26
TOMM70
SEC61A1
SEC22B
Protein interaction score
Aβ1-42 correlation
Pearson's coefficient
D
r = 0.76
p = 0.0115
BAG3
r = 0.78
p = 0.0084
DCD
r = 0.81
p = 0.0046
LRRFIP1
Retinal Aβ1-42
E
r = -0.77
p = 0.0154
TMT1A
r = -0.88
p = 0.0008
AP2A2
F
0.0889
0N4R TAU
NC
AD
G
r = 0.65
p = 0.0220
GSDMD
0N4R TAU
H
0N4R TAU correlation
Pearson's coefficient
I
r = 0.85
p = 0.0005
BAG3
r = 0.90
p < 0.0001
RAD23B
r = 0.81
p = 0.0015
LRRFIP1
r = 0.91
p < 0.0001
CAST
0N4R TAU
J
r = -0.92
p < 0.0001
TMT1A
0N4R TAU

Supplementary Figure 8. Correlation of *Chlamydia* inclusion interactors with amyloid plaque and neurofibrillary tangle burden in the AD retina.

A, B Pearson's correlation coefficient analysis of $A\beta_{1-42}$ levels with (**A**) Caspase-3 (Casp3) and (**B**) Gasdermin D (GSDMD). Retinal $A\beta_{1-42}$ was quantified by using sandwich enzyme-linked immunosorbent assay (ELISA) from human AD (n = 5) and NC retina (n = 5). **C** Protein interaction network (String v12.0) of *Chlamydia* interactors and their correlation with $A\beta_{1-42}$ levels. Node size represents the Pearson's correlation coefficient (*r*). Dark green and orange node borders indicate negative and positive correlation with $A\beta_{1-42}$ levels, respectively. Inner ring categorizes the protein's role into apoptosis (blue), immune response (grey) and/or cytokine signaling (olive). The thickness of edges between nodes represents the protein interaction score from String v12.0. Correlation statistics: unadjusted ⋆$p<0.05$, ⋆⋆$p<0.01$, and ⋆⋆⋆$p<0.001$. **D, E** Pearson's correlation coefficients analysis between several *Chlamydia* interactors and retinal $A\beta_{1-42}$ levels. Interactors with strongest (**D**) positive and (**E**) negative correlations are shown. **F** Mass spectrometry quantitation of 0N4R isoform of TAU [absence of N-terminal insert (0N) and presence of four microtubule-binding repeat domains (4R)] in human AD (n = 6) and NC retina (n = 6). *P* value determined by two-sided *t*-test. **G** Pearson's correlation coefficient analysis of 0N4R TAU with GSDMD. **H** The same protein interaction network of *Chlamydia* interactors showing their correlation to 0N4R TAU. Node size represents the Pearson's correlation coefficient (*r*). Dark green and orange node borders indicate negative and positive correlation with 0N4R TAU, respectively. Inner ring categorizes the protein's role in apoptosis (blue), immune response (grey), and/or cytokine signaling (olive). The thickness of edges between nodes represents the protein interaction score from String v12.0. Correlation statistics: ⋆$p<0.05$, ⋆⋆$p<0.01$, ⋆⋆⋆$p<0.001$, and ⋆⋆⋆⋆$p<0.0001$. **I, J** Pearson's correlation coefficients analysis between several *Chlamydia* interactors and 0N4R TAU quantitated in the retina by MS. Interactors with strongest (**I**) positive and (**J**) negative correlations are shown. Source data are provided as a Source Data file.

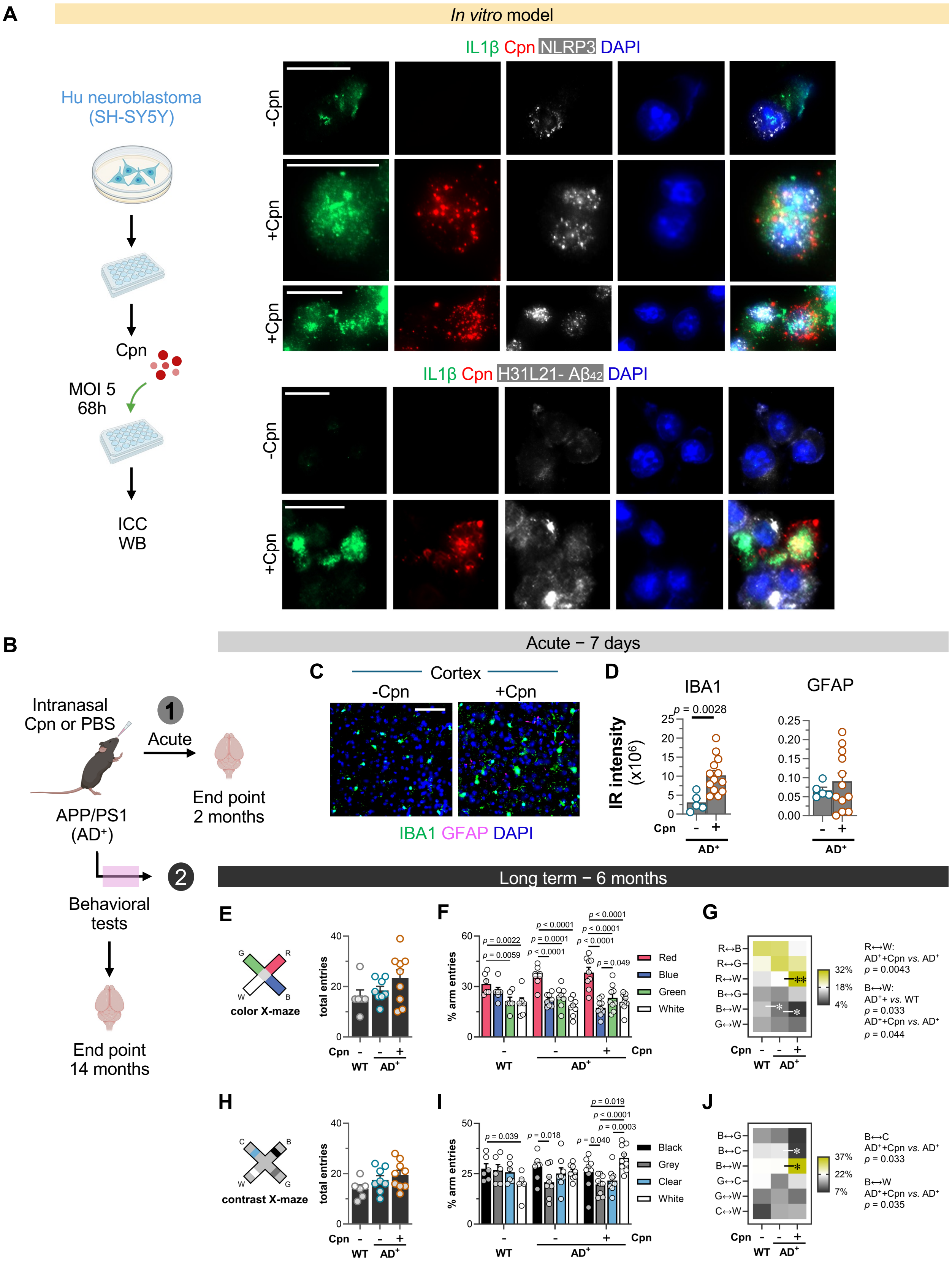

A
In vitro model
IL1β Cpn NLRP3 DAPI
Hu neuroblastoma (SH-SY5Y)
-Cpn
+Cpn
+Cpn
Cpn
MOI 5
68h
ICC
WB
IL1β Cpn H31L21- Aβ42 DAPI
-Cpn
+Cpn
B
Acute – 7 days
Intranasal Cpn or PBS
1
Acute
End point 2 months
APP/PS1 (AD+)
2
Behavioral tests
End point 14 months
C
Cortex
-Cpn
+Cpn
IBA1 GFAP DAPI
D
IBA1
GFAP
IR intensity (x10^6)
p = 0.0028
Cpn
AD+
Long term – 6 months
E
color X-maze
total entries
WT AD+
F
% arm entries
Red
Blue
Green
White
G
R↔W: AD+Cpn vs. AD+ p = 0.0043
B↔W: AD+ vs. WT p = 0.033 AD+Cpn vs. AD+ p = 0.044
H
contrast X-maze
I
Black
Grey
Clear
White
J
B↔C AD+Cpn vs. AD+ p = 0.033
B↔W AD+Cpn vs. AD+ p = 0.035

Supplementary Figure 9. Effects of Cpn infection in human neuroblastoma (SH-SY5Y) cells and AD transgenic (AD^+) mice.

A Experimental scheme and representative fluorescence micrographs of human SH-SY5Y neuroblastoma immunolabeled for IL1β (green), *Chlamydia pneumoniae* (Cpn; red), NLRP3 or $Aβ_{42}$ (clone H31L21; white), DAPI (blue). Scale bars, 20 µm. **B** Schematic illustration of the *in vivo* study in double transgenic $APP_{SWE}/PS1_{ΔE9}$ (AD^+) mice. **C** Representative fluorescence micrographs of cortical regions immunolabeled for IBA1 microglia (green), GFAP astrocytes (magenta), and nuclei (DAPI, blue) in the acute, 7-day infection model of AD^+ mice ①. Scale bar, 50 µm. **D** Quantitative analyses of IBA1 (−Cpn = 5, +Cpn = 13) and GFAP (−Cpn = 5, +Cpn = 12) immunoreactive intensity in the cortex of AD^+ mice. **E-J** Behavioral assessments in the long-term, 6-month infection model of AD^+ mice ② (WT = 6, AD^+-Cp = 8, AD^++Cp = 9), using the X-maze, showing total number of arm entries (all arms), percentage of arm entries (each arm), and bidirectional transitions in (**E-G**) color mode and (**H-J**) contrast mode. Statistics: Data from individual subjects (circles) and group means ± SEMs are shown. *p* values are by one- or two-way ANOVA with Fisher's LSD post hoc multiple-comparison test, or by Mann-Whitney U test. Source data are provided as a Source Data file. Illustrations A and B were created in Biorender.com.

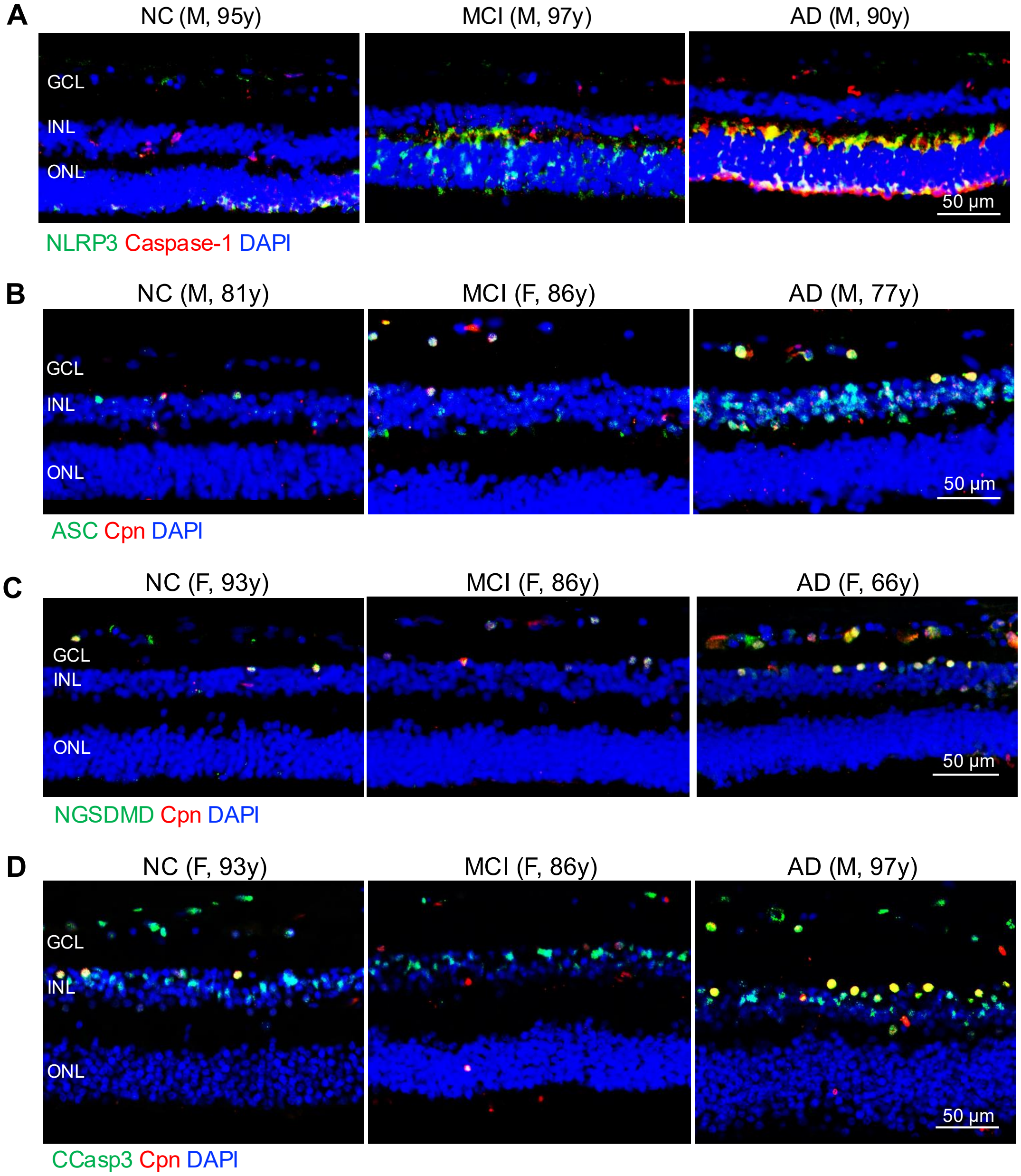

Supplementary Figure 10. Extended images of retinal NLRP3 inflammasome components, early apoptosis, and cellular pyroptosis markers.

A-D Representative images of retinal cross-sections from MCI and AD patients versus NC controls stained with **(A)** NLRP3 (green) and Caspase-1, (red), (**B**) ASC (green) and *Chlamydia pneumoniae* (Cpn; red), (**C**) N-terminal cleaved gasdermin-D (NGSDMD; green) and Cpn (red), (**D**) cleaved caspase-3 (CCasp3; green) and Cpn (red), with DAPI (blue) nuclear staining. Colocalization are shown in yellow. M-male, F-female, and age (y) are shown. Scale bars, 50 µm.

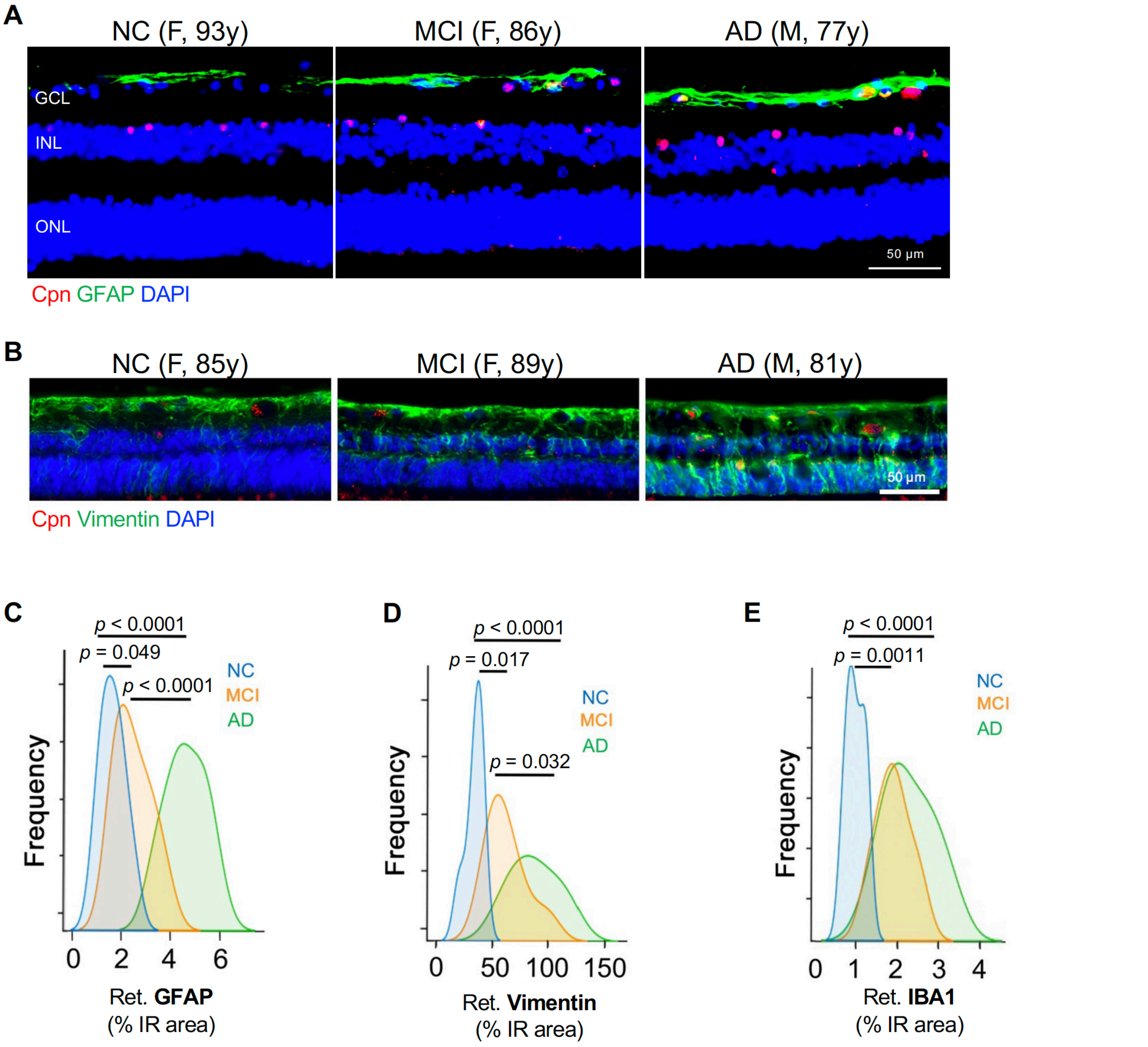

Supplementary Figure 11. Extended data on retinal gliosis and Cpn.

A, B Representative images of retinal cross-sections from MCI and AD patients versus NC controls stained with (**A**) *Chlamydia pneumoniae* (Cpn; red) and GFAP (green) and (**B**) Cpn (red) and vimentin (green), with DAPI (blue) nuclear staining. Scale bars, 50 µm. **C-E** Gaussian distribution curves displaying the frequency of (**C**) retinal GFAP^{+} macrogliosis % IR area in donors with NC (n = 8), MCI (n = 10), and AD dementia (n = 10), (**D**) retinal Müller glia marker (vimentin; % IR area) in donors with NC (n = 6), MCI (n = 8), and AD (n = 7), and (**E**) retinal IBA1 % IR area in donors with NC (n = 9), MCI (n = 9), and AD (n = 14). M-male, F-female, and age (y) are shown. Statistics: Data from individual subjects (circles) as well as group means ± SEMs are shown. *p* values are by one-way ANOVA and Tukey's post hoc multiple comparison test. Source data are provided as a Source Data file.

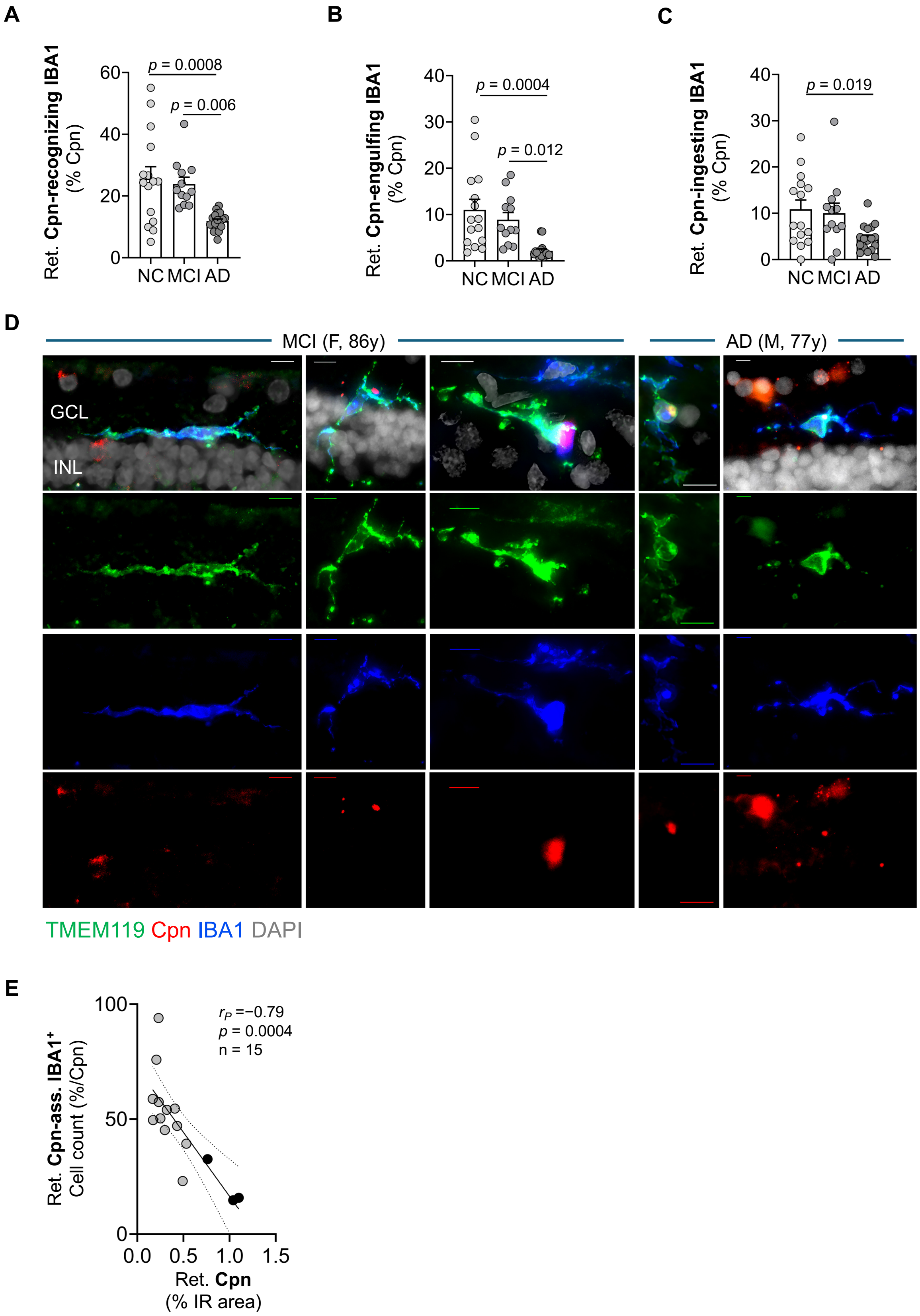
A
Ret. Cpn-recognizing IBA1 (% Cpn)
p = 0.0008
p = 0.006
NC MCI AD
B
Ret. Cpn-engulfing IBA1 (% Cpn)
p = 0.0004
p = 0.012
NC MCI AD
C
Ret. Cpn-ingesting IBA1 (% Cpn)
p = 0.019
NC MCI AD
D
MCI (F, 86y)
AD (M, 77y)
GCL
INL
TMEM119 Cpn IBA1 DAPI
E
Ret. Cpn-ass. IBA1+ Cell count (%/Cpn)
rP =−0.79
p = 0.0004
n = 15
Ret. Cpn (% IR area)

Supplementary Figure 12. Cpn-associated microglia in retinas from AD, MCI, and NC individuals. **A-C** Scatter plots of quantitative IHC analysis for retinal microgliosis (IBA1) per retinal Cp % IR area in three microglial stages for *Chlamydia pneumoniae* (Cpn; red) phagocytosis (**A**) Cpn-recognizing microglia, (**B**) Cpn-engulfing microglia, and (**C**) Cpn-ingesting microglia in individuals with premortem clinical diagnoses of NC (n = 15), MCI (n = 12), and AD (n = 17). **D** Fluorescence micrographs showing microglia stained with TMEM119 (green) and IBA1 (blue) recognizing, engulfing, or uptaking/ingesting Cpn (red). Nuclei are shown in grey. Scale bars, 10 µm. **E** Pearson's (r_P) correlation analysis between retinal Cpn load and Cpn-associated microglia in the NC group only. Black dots indicate 3 individuals with high retinal Cpn-burden. M-male, F-female, and age (y) are shown. Statistics: Data from individual subjects (circles) and group means ± SEMs are shown. *p* values are by one-way ANOVA and Tukey's post hoc multiple comparison test. Source data are provided as a Source Data file.

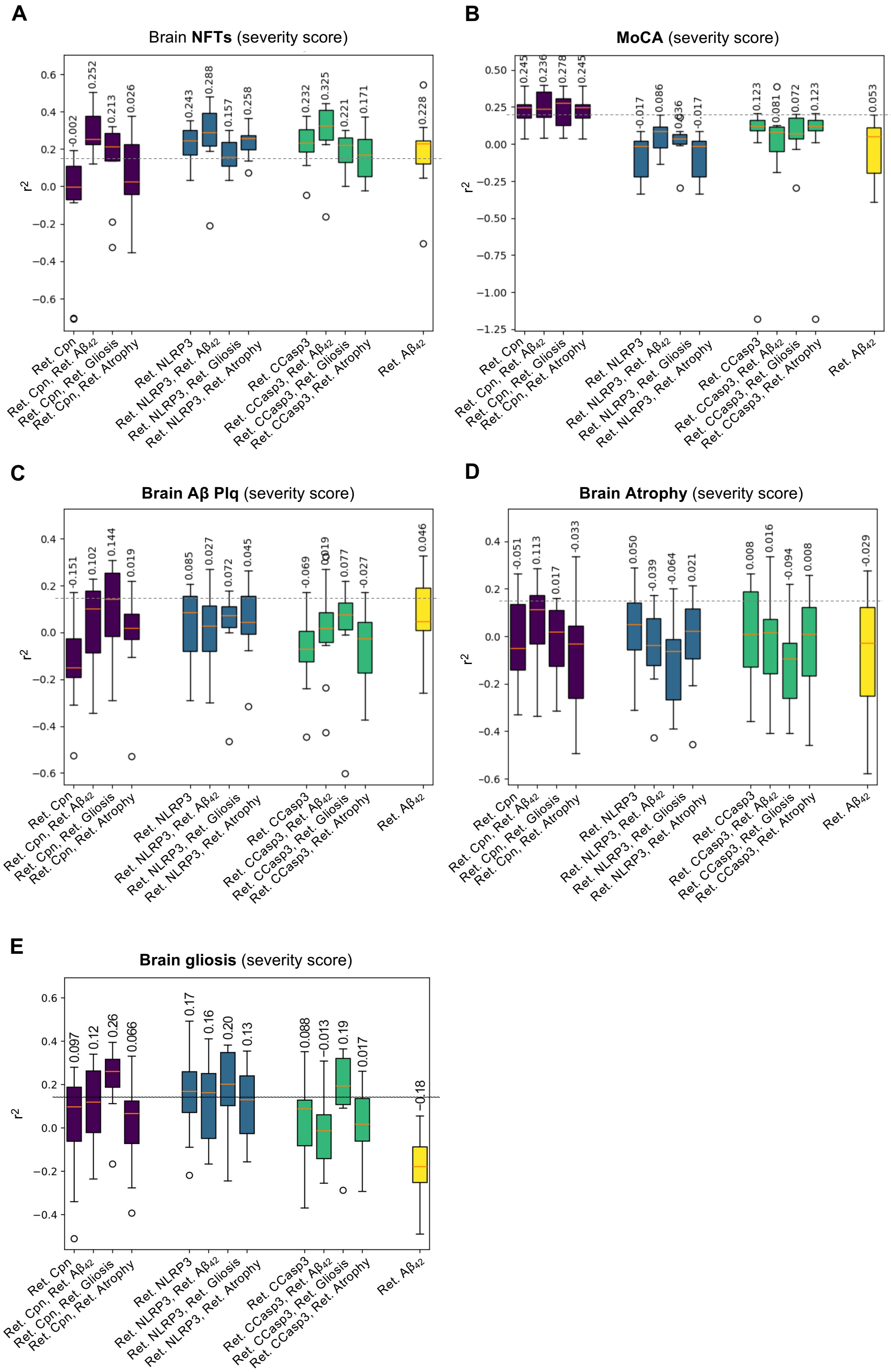
A
Brain NFTs (severity score)
B
MoCA (severity score)
C
Brain Aβ Plq (severity score)
D
Brain Atrophy (severity score)
E
Brain gliosis (severity score)
r2
Ret. Cpn
Ret. Cpn, Ret. Aβ42
Ret. Cpn, Ret. Gliosis
Ret. Cpn, Ret. Atrophy
Ret. NLRP3
Ret. NLRP3, Ret. Aβ42
Ret. NLRP3, Ret. Gliosis
Ret. NLRP3, Ret. Atrophy
Ret. CCasp3
Ret. CCasp3, Ret. Aβ42
Ret. CCasp3, Ret. Gliosis
Ret. CCasp3, Ret. Atrophy
Ret. Aβ42

Supplementary Figure 13. Prediction of brain AD pathologies by retinal *Chlamydia pneumoniae*, NLRP3, cleaved caspase-3, and Aβ_{42}.

A-E Machine learning algorithm with a Random Forest regressor using 80 estimators was trained on the data to predict several brain pathologies and cognitive status, including (**A**) brain NFT severity score (n=24/25 for train/validation), (**B**) Montreal cognitive assessment (MOCA) score (n=6/6 for train/validation), (**C**) total brain Aβ plaques severity score (n=24/25 for train/validation), (**D**) brain atrophy severity score (n=24/25 for train/validation), and (**E**) brain gliosis (n=24/25 for train/validation) by retinal *Chlamydia pneumoniae* (Cpn), NLRP3, CCasp3, or Aβ_{42}, either individually or combined with retinal Aβ_{42}, or retinal gliosis (IBA1, GFAP, and Vimentin), or retinal atrophy index. The distributions show the spread of models trained on different folds of the 5x2 cross-validation. The mean r^2 for each model is shown at the top of each box plot. Only models performing with variance coefficient $r^2 > 0.15$ were retained. Source data are provided as a Source Data file.

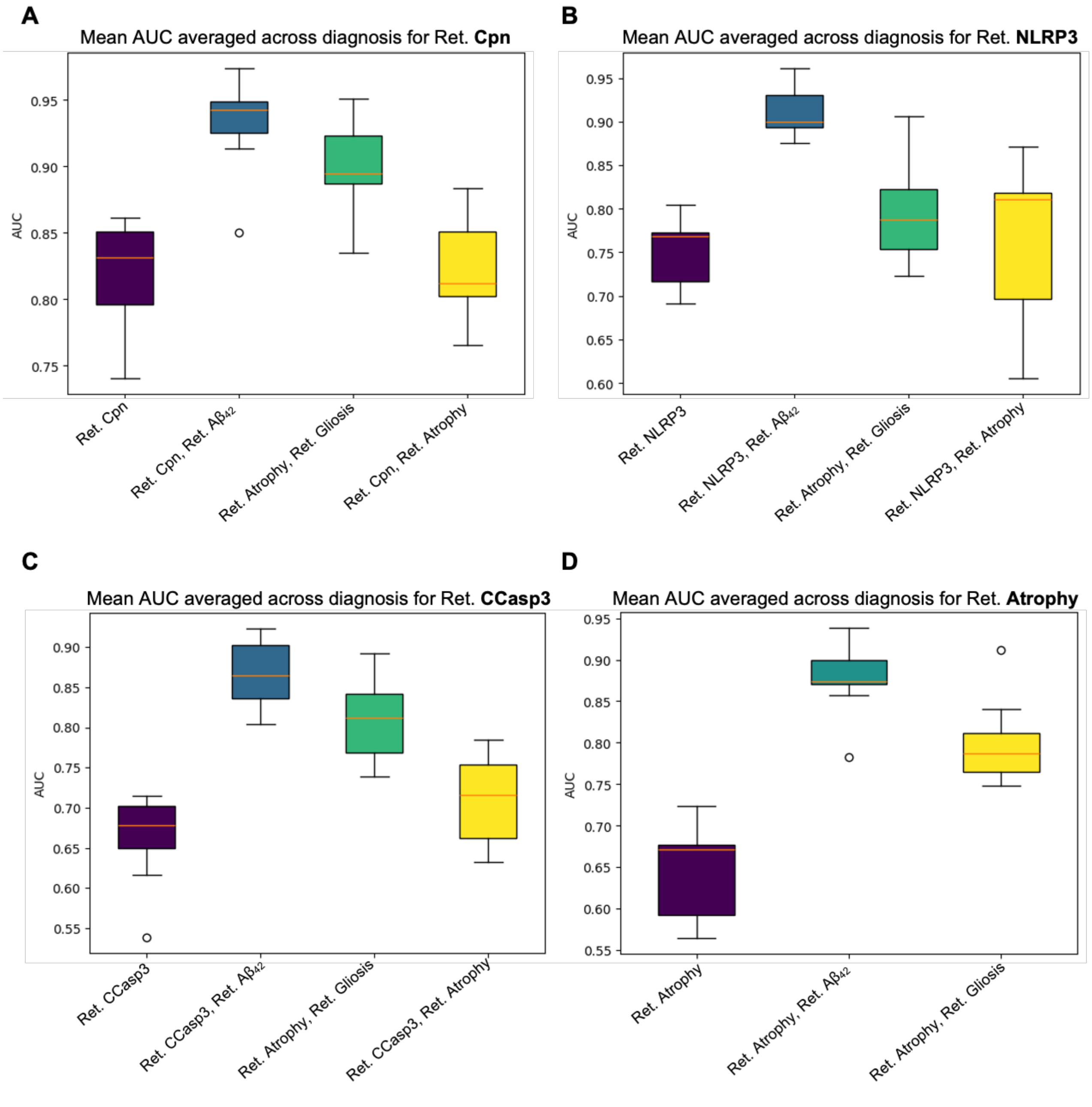

Supplementary Figure 14. The AUC box plots for retinal biomarkers across all diagnostic groups.

A-D Box plots representing the AUC measure for (**A**) retinal *Chlamydia pneumoniae* (Cpn), (**B**) retinal NLRP3, (**C**) retinal CCaspase3, and (**D**) retinal atrophy index for all diagnostic groups combined. For each model, AUC was measured either individually or combined with retinal Aβ_{42}, or retinal gliosis (IBA1, GFAP, and Vimentin), or retinal atrophy index. Source data are provided as a Source Data file.

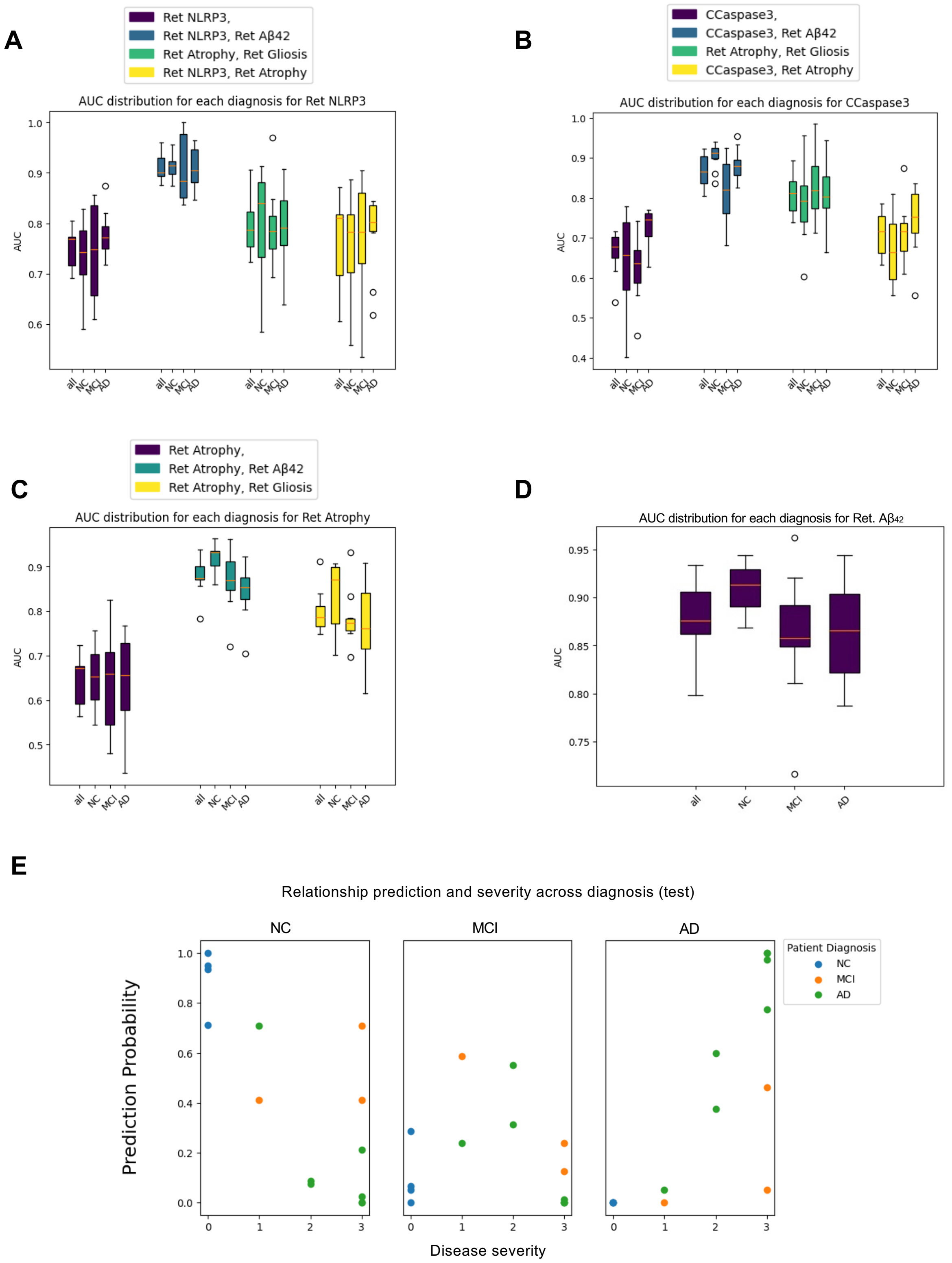
A
Ret NLRP3,
Ret NLRP3, Ret Aβ42
Ret Atrophy, Ret Gliosis
Ret NLRP3, Ret Atrophy
AUC distribution for each diagnosis for Ret NLRP3
AUC
all
NC
MCI
AD
B
CCaspase3,
CCaspase3, Ret Aβ42
Ret Atrophy, Ret Gliosis
CCaspase3, Ret Atrophy
AUC distribution for each diagnosis for CCaspase3
C
Ret Atrophy,
Ret Atrophy, Ret Aβ42
Ret Atrophy, Ret Gliosis
AUC distribution for each diagnosis for Ret Atrophy
D
AUC distribution for each diagnosis for Ret. Aβ42
E
Relationship prediction and severity across diagnosis (test)
NC
MCI
AD
Patient Diagnosis
Prediction Probability
Disease severity

Supplementary Figure 15. The AUC box plots for retinal biomarkers across each diagnostic groups and prediction of disease severity.

Random forest regressor using 80 estimators was trained on the data to predict brain gliosis by retinal *Chlamydia pneumoniae* (Cpn) alone or in combination with retinal NLRP3, CCasp3, or Aβ_{42}. **A-D** Box plots representing the AUC measure for (**A**) retinal NLRP3, (**B**) retinal CCasp3, and (**C**) retinal atrophy, alone or in combination with other retinal markers, for all and each diagnostic group. For each model, AUC was measured either individually or combined with retinal Aβ_{42}, or retinal gliosis (IBA1, GFAP, and Vimentin), or retinal atrophy index. **D** Box plots representing the AUC measure for retinal Aβ_{42} for all and each diagnostic groups NC, MCI and AD. **E** Plots showing retinal Cpn prediction probabilties for each subject in the test set and their relation to disease severity. Each individual is colored by their true label. MCI patients (orange) were rated closer to the average (1/3) prediction when compared to either normal cognition or AD patients, indicating uncertainty in the model's prediction. AD patients had AD predictions that were proportional to their disease severity, while normal cognition predictions were inversely proportional to disease severity. Source data are provided as a Source Data file.

References:

1. Koronyo Y, Rentsendorj A, Mirzaei N, et al. Retinal pathological features and proteome signatures of Alzheimer's disease. *Acta Neuropathol*. Apr 2023;145(4):409-438. doi:10.1007/s00401-023-02548-2
2. Aeberhard L, Banhart S, Fischer M, et al. The Proteome of the Isolated Chlamydia trachomatis Containing Vacuole Reveals a Complex Trafficking Platform Enriched for Retromer Components. *PLoS Pathog*. Jun 2015;11(6):e1004883. doi:10.1371/journal.ppat.1004883
3. Dickinson MS, Anderson LN, Webb-Robertson BM, et al. Proximity-dependent proteomics of the Chlamydia trachomatis inclusion membrane reveals functional interactions with endoplasmic reticulum exit sites. *PLoS Pathog*. Apr 2019;15(4):e1007698. doi:10.1371/journal.ppat.1007698
4. Olson MG, Widner RE, Jorgenson LM, et al. Proximity Labeling To Map Host-Pathogen Interactions at the Membrane of a Bacterium-Containing Vacuole in Chlamydia trachomatis-Infected Human Cells. *Infect Immun*. Nov 2019;87(11)doi:10.1128/IAI.00537-19
5. Mirrashidi KM, Elwell CA, Verschueren E, et al. Global Mapping of the Inc-Human Interactome Reveals that Retromer Restricts Chlamydia Infection. *Cell Host Microbe*. Jul 8 2015;18(1):109-21. doi:10.1016/j.chom.2015.06.004
6. Olson MG, Ouellette SP, Rucks EA. A meta-analysis of affinity purification-mass spectrometry experimental systems used to identify eukaryotic and chlamydial proteins at the Chlamydia trachomatis inclusion membrane. *J Proteomics*. Feb 10 2020;212:103595. doi:10.1016/j.jprot.2019.103595
7. Shi H, Mirzaei N, Koronyo Y, et al. Identification of retinal oligomeric, citrullinated, and other tau isoforms in early and advanced AD and relations to disease status. *Acta Neuropathol*. Jul 9 2024;148(1):3. doi:10.1007/s00401-024-02760-8
