# Supplementary material for: Identification of Chlamydia pneumoniae and NLRP3 inflammasome activation in Alzheimer’s disease retina
Source: Nat Commun. 2026 Jan 22;17:771. doi: 10.1038/s41467-026-68580-4 (PMC12827417; doi:10.1038/s41467-026-68580-4)
Supplement: Supplementary file 2 — Reporting Summary [file 41467_2026_68580_MOESM2_ESM.pdf]

nature portfolio

Corresponding author(s): Maya Koronyo-Hamaoui, Timothy R. Crother

Last updated by author(s): 12/23/2025

# Reporting Summary

Nature Portfolio wishes to improve the reproducibility of the work that we publish. This form provides structure for consistency and transparency in reporting. For further information on Nature Portfolio policies, see our Editorial Policies and the Editorial Policy Checklist.

## Statistics

For all statistical analyses, confirm that the following items are present in the figure legend, table legend, main text, or Methods section.

| n/a | Confirmed | |
|---|---|---|
| ☐ | ☒ | The exact sample size (*n*) for each experimental group/condition, given as a discrete number and unit of measurement |
| ☐ | ☒ | A statement on whether measurements were taken from distinct samples or whether the same sample was measured repeatedly |
| ☐ | ☒ | The statistical test(s) used AND whether they are one- or two-sided<br>*Only common tests should be described solely by name; describe more complex techniques in the Methods section.* |
| ☐ | ☒ | A description of all covariates tested |
| ☐ | ☒ | A description of any assumptions or corrections, such as tests of normality and adjustment for multiple comparisons |
| ☐ | ☒ | A full description of the statistical parameters including central tendency (e.g. means) or other basic estimates (e.g. regression coefficient) AND variation (e.g. standard deviation) or associated estimates of uncertainty (e.g. confidence intervals) |
| ☐ | ☒ | For null hypothesis testing, the test statistic (e.g. *F*, *t*, *r*) with confidence intervals, effect sizes, degrees of freedom and *P* value noted<br>*Give P values as exact values whenever suitable.* |
| ☒ | ☐ | For Bayesian analysis, information on the choice of priors and Markov chain Monte Carlo settings |
| ☐ | ☒ | For hierarchical and complex designs, identification of the appropriate level for tests and full reporting of outcomes |
| ☐ | ☒ | Estimates of effect sizes (e.g. Cohen's *d*, Pearson's *r*), indicating how they were calculated |

*Our web collection on statistics for biologists contains articles on many of the points above.*

## Software and code

Policy information about availability of computer code

| | |
|---|---|
| Data collection | 1- Microscopy: Carl Zeiss Axio Imager Z1 fluorescence and brightfield microscope with ZEN 2.6 blue edition software (Carl Zeiss MicroImaging, Inc.).<br>2- Western blot: LI-COR Odyssey imaging system.<br>3- Proteomics (Mass spectrometry, MS): Cleaned peptides from each fraction were collected and analyzed using a Q Exactive Orbitrap mass spectrometer (MS; Thermo Scientific) coupled to an EASY-nLC1000 nanoflow HPLC system (Thermo Scientific).<br>4- ELISA: SpectraMax M2 (molecular devices).<br>5- qPCR analysis: CFX96 Real-Time System (BioRad).<br>6- Mouse maze activity (video recording and tracking): ANY-maze behavior tracking software 6.3 (www.stoeltingco.com/anymaze/video-tracking/software.html) or other video tracking software, such as EthoVision XT (www.noldus.com/ethovision-xt).<br>7- Open Field mouse recording: Photobeam Activity System (www.sandiegoinstruments.com (http://www.sandiegoinstruments.com/) |
| Data analysis | 1- Proteomics: Proteome Discoverer V2.1 software (Thermo Scientific) and Mascot (Matrix Science, UK). We used Gene Ontology (GO) in Metascape (https://metascape.org/) and included the GO Biological Processes, Reactome, Kyoto Encyclopedia of Genes and Genomes (KEGG) and WikiPathways databases.<br>Cytoscape 3.10.2 (https://cytoscape.org/).<br>Protein interaction networks were generated using String v12.0 and Cytoscape.<br>2- Heatmaps were generated in ClustVis (https://biit.cs.ut.ee/clustvis/) and Prism 10.2.3 (GraphPad).<br>3- Chord diagrams were created in Circos online (https://mk.bcgsc.ca/tableviewer/).<br>4- Data collection and summary: Microsoft 365 Excel, PowerPoint and Word, Adobe Photoshop.<br>5- Statistical analysis: Prism 10.2.3 (GraphPad). |

6- Machine learning: The data were processed, and models were trained using a combination of Scikit-learn, Numpy, Pandas, Scipy, and custom Python 3.11 code (https://github.com/xomicsdatascience/Retinal_Alzheimer_Prediction). Random forest models were used for the prediction.
7- Histology: Image processing: ZEN 2.6 blue edition soft- ware; ImageJ2/Fiji (version 2.14.0; NIH).
8- Western blot: Image Studio software (LI-COR).
9- qPCR: Bio-Rad CFX Manager 3.1.
10- Rodent Toolbox V1 (freely available at https://www.ndcn.ox.ac.uk/team/stuart-peirson).
11- ViS4M Toolbox V1.
12- ParallelSets V2 (freely available at Parallel Sets (eagereyes.org)).
13- Circos online (freely available at mkweb.bcgsc.ca/tableviewer/).

For manuscripts utilizing custom algorithms or software that are central to the research but not yet described in published literature, software must be made available to editors and reviewers. We strongly encourage code deposition in a community repository (e.g. GitHub). See the Nature Portfolio guidelines for submitting code & software for further information.

## Data

Policy information about availability of data

All manuscripts must include a data availability statement. This statement should provide the following information, where applicable:

- Accession codes, unique identifiers, or web links for publicly available datasets
- A description of any restrictions on data availability
- For clinical datasets or third party data, please ensure that the statement adheres to our policy

- All data generated or analyzed for this study are included in this manuscript, supplementary material, and source data file attached with the submission.
- All data generated for multi-variable analyses are available on Zenodo at GitHub (https://github.com/xomicsdatascience/Retinal_Alzheimer_Prediction).
- All immunohistochemistry and proteomics data generated in this study have been included in the manuscript and the Online supplementary materials. The mass spectrometry raw files and search results have been deposited to the ProteomeXchange Consortium via the PRIDE partner repository with the dataset identifier PXD040225.

## Research involving human participants, their data, or biological material

Policy information about studies with human participants or human data. See also policy information about sex, gender (identity/presentation), and sexual orientation and race, ethnicity and racism.

| | |
|---|---|
| Reporting on sex and gender | Human postmortem donor tissues of both sexes were analyzed, with comparable female-to-male ratios across diagnostic groups. The retinal and brain tissues (N=104) were collected from clinically and neuropathologically confirmed mild-cognitive impaired (MCIdue to AD) and Alzheimer's disease (AD) patients, and age- and sex-matched individuals with normal cognition (Demographic data are detailed in Table 1 and Supplementary Tables 1-5). |
| Reporting on race, ethnicity, or other socially relevant groupings | Among 104 human donors used in this study, most of them were white (83%). Other ethnic groups were Black (2%), Hispanic (12%), and Asian (3%) per availability (see Table 1 and Supplementary Tables 1-3). |
| Population characteristics | The detailed information about age, sex, race, APOE ε4 genotype, diagnosis, and brain pathology are detailed in Table 1 and Supplementary Tables 1-5. |
| Recruitment | Not applicable |
| Ethics oversight | Collection of postmortem brain and eye globes were in accordance with the institutional ethics protocols: Alzheimer’s Disease Research Center (ADRC) Neuropathology Core at the Department of Pathology in the University of Southern California (USC, Los Angeles, CA; IRB protocol HS-042071); National Disease Research Interchange (NDRI, Philadelphia, PA; under Cedars-Sinai IRB protocol Pro00019393); ADRC Neuropathology Core at the University of California, Irvine (UCI IRB protocol HS#2014–1526); the Rush Alzheimer’s Disease Center (RADC) at Rush University (Chicago, IL; ORA# 18011111). All the histological procedures were conducted at Cedars-Sinai Medical Center under IRB protocols (Pro00053412, Pro00019393, and Pro00055802). |

Note that full information on the approval of the study protocol must also be provided in the manuscript.

# Field-specific reporting

Please select the one below that is the best fit for your research. If you are not sure, read the appropriate sections before making your selection.

[x] Life sciences [ ] Behavioural & social sciences [ ] Ecological, evolutionary & environmental sciences

For a reference copy of the document with all sections, see nature.com/documents/nr-reporting-summary-flat.pdf

# Life sciences study design

All studies must disclose on these points even when the disclosure is negative.

| | |
|---|---|
| Sample size | Sample sizes for comparisons of two groups (differential mean) were calculated using nQUERY t-test model, assuming a two-sided alpha level of 0.05, 80% power and unequal variances, with the means and common standard deviations for the different parameters. |
| Data exclusions | Statistical analysis: All analyzed data are included and presented in the manuscript. No data were excluded, except for one outlier (>2 standard deviations) in Supplementary Figure 5A–B.<br>In terms of human donors, tissues were collected from 3 diagnostic groups: 1. confirmed AD dementia, 2. MCI due to AD, and 3. cognitively normal. Subjects with macular degeneration, diabetic retinopathy, and glaucoma were excluded. |
| Replication | Immunohistological analyses, RT–PCR, and gDNA-qPCR assays were performed at least three times. Giemsa staining and FISH analyses were performed at least three times. Western blot and cell culture experiments were repeated twice. Machine-learning analyses were conducted five times. In vivo animal studies included two experiments: acute (7 days post-infection cohort) and long-term (6 months post-infection cohort) with experimental group sizes of at least 5 animals/group (repeated twice). All experiments showed consistent and reproducible results. |
| Randomization | Postmortem retinal and brain tissues were obtained from clinically and neuropathologically confirmed individuals with mild cognitive impairment (MCI, due to AD) and Alzheimer's disease (AD) dementia, as well as from cognitively normal individuals (age- and sex-matched controls), all classified according to Alzheimer's disease neuropathologic change (ADNC) criteria. Samples were randomly selected to achieve balanced group sizes and stratified based on APOE ε4 genotype, neurofibrillary tangle (NFT) burden, Braak stage, cognitive assessments (MMSE, CDR, MOCA), and sex. This randomization strategy ensured robust group comparisons for evaluating Chlamydia pneumoniae (Cpn) burden and related NLRP3 inflammasome activation components in relation to disease status. All histological, in vivo, and in vitro experiments were randomized, and investigators were blinded during allocation, experimentation, and outcome assessments. |
| Blinding | Throughout the IHC and mass spectrometry analyses, researchers were blinded to patient diagnosis and animal genotype.<br>For animal behavior, mice and cages were coded so that the experimenter was blinded to genotype and treatment.<br>For the in vitro studies, cell culture well were randomly assigned to infected or non-infected group and subsequent analyses were performed blindly as much as possible.<br>Blinding was not applicable for ELISA, qPCR, as their quantification is instrument-based and does not involve observer-dependent scoring. |

# Reporting for specific materials, systems and methods

We require information from authors about some types of materials, experimental systems and methods used in many studies. Here, indicate whether each material, system or method listed is relevant to your study. If you are not sure if a list item applies to your research, read the appropriate section before selecting a response.

## Materials & experimental systems

| n/a | Involved in the study | |
|---|---|---|
| ☐ | ☒ | Antibodies |
| ☐ | ☒ | Eukaryotic cell lines |
| ☒ | ☐ | Palaeontology and archaeology |
| ☐ | ☒ | Animals and other organisms |
| ☐ | ☒ | Clinical data |
| ☒ | ☐ | Dual use research of concern |
| ☒ | ☐ | Plants |

## Methods

| n/a | Involved in the study | |
|---|---|---|
| ☒ | ☐ | ChIP-seq |
| ☒ | ☐ | Flow cytometry |
| ☒ | ☐ | MRI-based neuroimaging |

## Antibodies

| | |
|---|---|
| Antibodies used | 1- Immunohistochemistry:<br>Chlamydia pneumoniae (Cpn) polyclonal (1:500, MyBioSource, catalog # MBS534621), Cpn monoclonal (1:50 and 1:200, Invitrogen, catalog # MA5-18183), NLRP3 monoclonal (1:100, R&D Systems, catalog # MAB7578), Caspase-1 monoclonal (1:150, R&D Systems, catalog # MAB62156), ASC (1:300, rabbit, ADIPOGEN, Catalog #AG-25B-0006), N-terminal GSDMD (1:500, Cell Signaling, catalog # 36425), cleaved Caspase-3 polyclonal (1:400, Cell Signaling, catalog # 9661), GFAP polyclonal (1:500, Invitrogen, catalog # 13-0300), IBA1 monoclonal (1:400, Wako, catalog # 019-19741), IBA1 monoclonal (1:300, novusbio, catalog # NB100-1028), Aβ (6E10) monoclonal antibody (Biolegend, catalog # 803001), (H31L21) Aβ42 monoclonal (1:250, ThermoFisher, catalog # 700254), Vimentin (1:350, Abcam, catalog # Ab92547), NeuN monoclonal (1:500, Abcam, catalog # Ab177487), Aβ oligomers (scFvA13) monoclonal antibody (1:450, obtained from Dr. Giovanni Meli), CitR209tau monoclonal antibody (1:5000, obtained from Dr. Daniel Lee), Phospho-tau (Ser396) polyclonal (1:500, Anaspec catalog # AS-54977), Phospho-tau (Ser202, Thr205, AT8) monoclonal antibody (1:250, ThermoFisher catalog # MN1020), MC-1 monoclonal antibody (1:200, obtained from late Dr. Peter Davies), Oligomeric tau monoclonal antibody (1:200, obtained from Rakez Kayed), and PHF-tau monoclonal antibody (1:200, obtained from late Dr. Peter Davies).<br>Secondary antibodies used: Cy3-conjugated anti-mouse and anti-rat (1:200, Jackson ImmunoResearch Laboratories, catalog # 715-165-150 and 712-165-153), Cy5-conjugated anti-rabbit (1:200, Jackson ImmunoResearch Laboratories, catalog # 711-175-152), Cy2-conjugated anti-goat (1:200, Jackson ImmunoResearch Laboratories), and HRP-conjugated anti-mouse and anti-rabbit antibodies for IHC/DAB (Vectastain Elite ABC Kit, catalog # PK-6102,and PK-6101). |

2- Western blot: anti-IL1beta (1:1000, Abcam, catalog #ab9722), Aβ42 (12F4, 1:1000, Biolegend, catalog #805501), NLRP3 (1:500, NovusBio, Catalog # NBP2-12446), NLRP3 (1:1000, ThermoFisher, catalog #MA5-23919), NGSDMD (1:1000, Cell Signaling, catalog #36425), GSDMD (1:1000, MyBiosource, catalog #MBS9613058), anti-GAPDH (1: 1000, Millipore Sigma, catalog #G8795), anti-GAPDH (1: 1000, Cell signaling, catalog #5174), beta-actin (1: 1000, Cell signaling, 3700S), IRDYE® 680RD (1:10000, Licorbio, 926-68071, 926-68070), IRDYE® 800CW (1:10000, Licorbio, 926-32211, 926-32210), Peroxidase affinipure® (anti-rat, anti-rabbit, 1:10000, Jackson ImmunoResearch Laboratories, catalog # 112-035-003, 111-035-003) antibodies.
We included detailed antibody information in Supplementary Table 6 of the manuscript.

| | |
|---|---|
| Validation | Antibodies listed above were validated for species reactivity, antigen, and application (peroxidase-based immunohistochemistry, fluorescence-based immunolabeling, and Western blot analysis) by the corresponding manufacturer. For example, Chlamydia pneumoniae (Cpn) monoclonal antibody (K83A) can only label Cpn and no other Chlamydia species (C. trachomatis and C. psittaci) or uninfected human cells (Invitrogen manufacturer). Specific usages are detailed in the manuscript and supplementary materials. Furthermore, all antibodies were assessed with control tissues including human/mouse brain or human retinal tissues, prior to use in experimentation. Routine controls were processed using identical protocols while omitting the primary antibodies to assess nonspecific labeling. |

# Eukaryotic cell lines

Policy information about cell lines and Sex and Gender in Research

| | |
|---|---|
| Cell line source(s) | SH-SY5Y human neuroblastoma cells (CRL-2266TM, ATCC). |
| Authentication | Commercially obtained SH-SY5Y cell line is authenticated by the supplier (ATCC), and no further authentication was performed by the authors. |
| Mycoplasma contamination | SH-SY5Y cells were routinely checked for mycoplasma contamination. No contamination occurred during the experiments. |
| Commonly misidentified lines (See ICLAC register) | not used |

# Animals and other research organisms

Policy information about studies involving animals; ARRIVE guidelines recommended for reporting animal research, and Sex and Gender in Research

| | |
|---|---|
| Laboratory animals | Chlamydia pneumoniae (Cpn, CM-1 strain; American Type Culture Collection-ATCC, Manassas, VA) infection experiments were conducted in double-transgenic B6.Cg-Tg(APPSWE/PS1ΔE9)85Dbo/Mmjax (RRID: MMRRC_034832-JAX) mice (AD$^+$) at 2 months of age (acute model) and 8 months of age (long-term model). Age-matched C57BL/6J wild-type mice were included as controls for the long-term infection studies. Animals were randomly assigned to infected or non-infected groups, with sex balanced as much as possible across groups. All mice were housed under identical environmental conditions at Cedars-Sinai Medical Center vivarium under standardized conditions: a 14 hours light/10 hours dark cycle, ambient temperature maintained at 74°F (23°C) ±2°F, and relative humidity at 30–70%, with ad libitum access to food and water and a maximum of five animals per cage. Prior to infection, mice were maintained in ventilated cages on Allentown IVC racks. Following infection or PBS treatment, mice were housed in ventilated cages on metro racks within negative-pressure cubicles. |
| Wild animals | Not applicable |
| Reporting on sex | Both male and female mice were used in this study. We did not observe significant sex-based differences in our findings so data from both sex were pooled for analyses. |
| Field-collected samples | not applicable |
| Ethics oversight | All procedures were approved by the Institutional Animal Care and Use Committee of Cedars-Sinai Medical Center (Los Angeles, CA) guidelines {approval number: IACUC008314 and IACUC 008475} and conducted in accordance with the NIH Guide for the Care and Use of Laboratory Animals and the ARRIVE guidelines. |

Note that full information on the approval of the study protocol must also be provided in the manuscript.

# Clinical data

Policy information about clinical studies
All manuscripts should comply with the ICMJE guidelines for publication of clinical research and a completed CONSORT checklist must be included with all submissions.

| | |
|---|---|
| Clinical trial registration | not applicable. There are no live human participants in this study. |
| Study protocol | not applicable |
| Data collection | not applicable |
| Outcomes | not applicable |

## Plants

| | |
|---|---|
| Seed stocks | not applicable |
| Novel plant genotypes | not applicable |
| Authentication | not applicable |
